# Discovery and multimerization of cross-reactive single-domain antibodies against SARS-like viruses to enhance potency and address emerging SARS-CoV-2 variants

**DOI:** 10.1038/s41598-023-40919-7

**Published:** 2023-08-22

**Authors:** Scott A. Hollingsworth, Cameron L. Noland, Karin Vroom, Anasuya Saha, Miranda Sam, Qinshan Gao, Haihong Zhou, David U. Grandy, Sujata Singh, Zhiyun Wen, Christopher Warren, Xiaohong Shirley Ma, Daniel Malashock, Jennifer Galli, Gwenny Go, Michael Eddins, Todd Mayhood, Karthik Sathiyamoorthy, Arthur Fridman, Fahimeh Raoufi, Yacob Gomez-Llorente, Andrea Patridge, Yinyan Tang, Shi-Juan Chen, Marc Bailly, Chengjie Ji, Laura J. Kingsley, Alan C. Cheng, Bernhard H. Geierstanger, Daniel M. Gorman, Lan Zhang, Kalyan Pande

**Affiliations:** 1 Computational and Structural Chemistry, Merck & Co., Inc., 213 East Grand Ave., South San Francisco, CA 94080 USA; 2 Discovery Biologics, Merck & Co., Inc., 213 East Grand Ave., South San Francisco, CA 94080 USA; 3 Infectious Disease and Vaccine Discovery, Merck & Co., Inc., 770 Sumneytown Pike, West Point, PA 19486 USA; 4 Data Science and Informatics, Merck & Co., Inc., 126 E. Lincoln Ave., Rahway, NJ 07065 USA; 5 NovaBioAssays, LLC, 52 Dragon Ct, Woburn, MA 01801 USA; 6 Present Address: Molecular Structure and Design, Bristol-Myers Squibb Research and Development, 700 Bay Road, Redwood City, CA 94063 USA; 7 Present Address: Boehringer Ingelheim, 900 Ridgebury Rd, Ridgefield, CT 06877 USA

**Keywords:** SARS-CoV-2, SARS virus, Biologics, X-ray crystallography

## Abstract

Coronaviruses have been the causative agent of three epidemics and pandemics in the past two decades, including the ongoing COVID-19 pandemic. A broadly-neutralizing coronavirus therapeutic is desirable not only to prevent and treat COVID-19, but also to provide protection for high-risk populations against future emergent coronaviruses. As all coronaviruses use spike proteins on the viral surface to enter the host cells, and these spike proteins share sequence and structural homology, we set out to discover cross-reactive biologic agents targeting the spike protein to block viral entry. Through llama immunization campaigns, we have identified single domain antibodies (VHHs) that are cross-reactive against multiple emergent coronaviruses (SARS-CoV, SARS-CoV-2, and MERS). Importantly, a number of these antibodies show sub-nanomolar potency towards all SARS-like viruses including emergent CoV-2 variants. We identified nine distinct epitopes on the spike protein targeted by these VHHs. Further, by engineering VHHs targeting distinct, conserved epitopes into multi-valent formats, we significantly enhanced their neutralization potencies compared to the corresponding VHH cocktails. We believe this approach is ideally suited to address both emerging SARS-CoV-2 variants during the current pandemic as well as potential future pandemics caused by SARS-like coronaviruses.

## Introduction

Coronaviruses (CoVs) are a large family of viruses that infect numerous species, including humans, and consist of four main genera known as alpha, beta, gamma, and delta. The most significant CoV species, severe acute respiratory syndrome coronavirus 2 (SARS-CoV-2), a beta-coronavirus that emerged in China in 2019, is currently driving a pandemic that has resulted in over 500 million cases and 15 million excess deaths in the last 3 years^1^. Other strains of CoV include SARS-CoV and MERS, both of which have resulted in smaller, but significant, outbreaks with high morbidity and mortality.

Coronaviruses are single-stranded RNA (ssRNA) viruses with a large genome size and a relatively high mutation rate. Recombination events among different CoV species have been shown to occur, resulting in further genetic variability^2^. One of the key factors driving the continued large burden of COVID-19 disease is the observed high rate of mutation of the SARS-CoV-2 virus, resulting in the emergence and rapid spread of novel viral variants capable of evading natural and vaccine-induced host immune responses. Based on systematic genomic sequencing of clinical isolates of SARS-COV-2, there are 12 lineage groups that have been identified and 5 of them, Alpha (B.1.1.7), Beta (B.1.351), Gamma (P.1), Delta (B.1.617.2), and the newly identified Omicron (B.1.1.529), have been defined as variants of concern (VOCs) by the World Health Organization. The Omicron variant, bearing more than 30 mutations in the viral spike protein, is currently the major variant circulating globally. Multiple studies have reported resistance of the Omicron variant against neutralization by antibodies and serum targeting the wild type (Wuhan) strain, significantly impacting the protective efficacy of original licensed Wuhan strain based vaccines and therapeutic antibodies^3–6^. Bivalent mRNA vaccines including the original Wuhan based strain and the currently circulating Omicron BA.4/5 strain were recently made available as booster vaccines, however, it is possible that new VOCs will arise and escape again from these boosters’ induced immunity. Furthermore, the threat of continued zoonotic spillovers warrants the development of broadly reactive antiviral agents that could combat coronaviruses with pandemic potential in the future^7^.

While the vast majority of antibodies targeting the SARS-CoV-2 spike protein have been conventional immunoglobulins, several potent heavy-chain variable domains (VHHs) from camelid-derived single-domain antibodies (sdAbs) targeting the CoV-2 spike have also been reported^8–10^. Several cross-reactive epitopes on the spike have been identified in the literature, including Class 3 and Class 4 epitopes on the receptor binding domain (RBD), represented by S309 and CR3022, respectively^11–13^. Since VHHs are smaller compared to conventional antibodies (~ 15 kDa vs ~ 150 kDa), they have the potential to bind to smaller, conserved epitopes shared among different coronaviruses that conventional antibodies might not access. Moreover, VHHs have been shown to possess favorable biophysical properties and their smaller size also facilitates generation of multivalent constructs. In this work, we report the discovery of multiple VHHs that bind distinct cross-reactive epitopes on the spike protein. We further demonstrate that the neutralization potencies of multimeric VHHs are greatly enhanced compared to the monovalent forms. The enhanced potency and potential to protect against escape mutations make this approach valuable in addressing emerging SARS-CoV-2 variants as well as SARS-like viruses that may emerge in the future.

## Results

### Identification of cross-reactive VHHs

**Figure 1:**
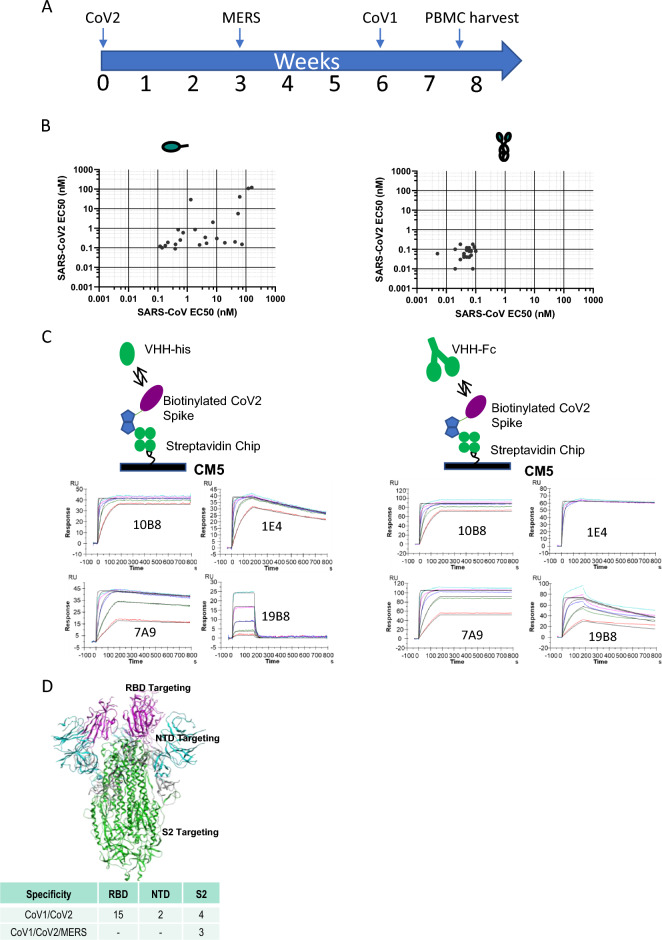
Identification of cross-reactive VHHs following llama immunization. (**A**) Llama immunization scheme using purified spike proteins from different viruses. (**B**) ELISA binding results of VHHs showing cross-binding to SARS-CoV and SARS-CoV-2 spike proteins (left panel, monovalent; right panel, bivalent). (**C**) SPR sensograms of select VHH binders to SARS-CoV-2 spike protein. Colored curves are raw data at different concentrations of VHH; black curves are fits to a 1:1 model. (**D**) Table of cross-reactive VHHs targeting different domains (RBD, NTD, S2) within the spike protein. The structure of the SARS-CoV-2 Spike (S) protein is shown (PDBID: 6XKL) and colored by domain; N-Terminal Domain (NTD) in cyan, Receptor Binding Domain (RBD) in pink, the S2 domain in green, and the stretch between the end of the RBD and beginning of the S2 domain colored gray.

We used a heterologous immunization approach to identify VHHs with broad cross-reactivity (Fig. 1A). We hypothesized that cross-reactive B cells would be enriched after three rounds of immunizations with related, but non-identical spike proteins. To that end, llamas were first immunized with SARS-CoV-2 spike protein, followed by spike proteins from MERS and SARS-CoV viruses with three-week intervals between the immunizations. Peripheral blood mononuclear cells (PBMCs) were harvested 10 days after the final immunization. B cells binding to SARS-CoV-2 spike protein were isolated and cultured, and supernatants were used to screen for binding to spike proteins from the three viruses. Twenty-three clones that bound to both SARS-CoV and SARS-CoV-2 spikes were sequenced, recombinantly expressed in either monomeric or bivalent formats, and validated for binding by ELISA to soluble recombinant spike proteins (Fig. 1B, Table S1). The binding by the bivalent constructs was also assessed by flow cytometry using cells recombinantly expressing spike protein on their surface (Table S2). All 23 constructs bound with similar affinity to SARS-CoV and SARS-CoV-2 with significant affinity improvement observed in the bivalent format, likely due to increased avidity. Many bivalent VHHs showed affinity to SARS-CoV-2 spike, and affinity mostly remained unchanged for SARS-CoV-2 variants Alpha, Beta, Gamma, and Delta. However, 8 of the candidates lost binding to the Omicron variants BA.1 and BA.2 (Table S1). In addition, one VHH (6A1) also bound to spike proteins from MERS and endemic beta human coronaviruses OC43 and HKU1 (Table S1). Direct affinity measurement against CoV-2 spike protein using Biacore (Fig. 1C, Table S3) validated ELISA results. Surface plasmon resonance (SPR) affinities were measured for monovalent VHHs. Monovalent affinities could not be measured for VHH-Fc constructs due to avidity, so the data do not fit the 1:1 model. The sensograms do, however, indicate that multivalent constructs had enhanced binding. Candidates were further evaluated for binding to different domains of the spike protein (RBD, NTD, S2) using ELISA (summarized in Fig. 1D). Using this approach, we were able to identify SARS-CoV and SARS-CoV-2 double cross-reactive clones targeting all 3 domains of the spike protein. In contrast, triple cross-reactive clones that bound to all three spike proteins (SARS-CoV, SARS-CoV-2, MERS) were restricted to the S2 domain.

### Potent cross-neutralization of SARS-CoV and SARS-CoV-2

**Figure 2:**
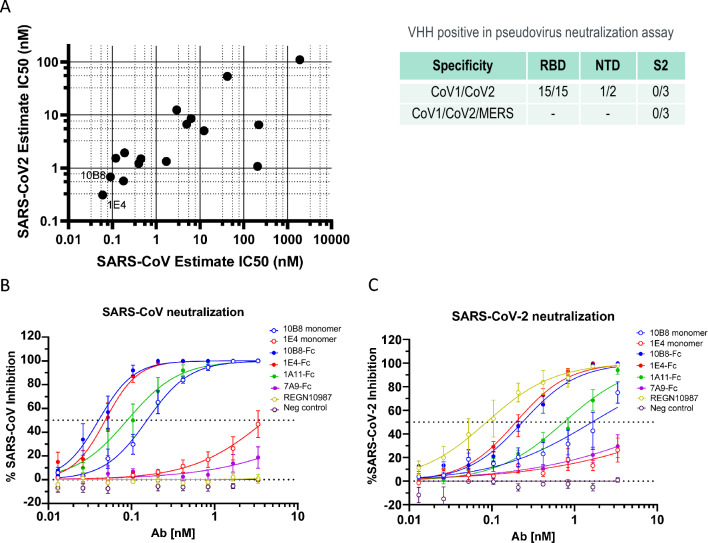
Pseudovirus and authentic virus neutralization. (**A**) SARS-CoV and SARS-CoV-2 VSV pseudovirus neutralization (estimated IC50 values) by 16 candidate VHH-Fcs (bivalent). (**B**) Authentic SARS-CoV virus neutralization by select candidates (monomeric and bivalent formats). (**C**) Authentic SARS-CoV-2 virus neutralization by select candidates (monomeric and bivalent formats).

We initially evaluated neutralization potency of candidate VHHs in the bivalent format using Vesicular stomatitis virus (VSV)-based pseudovirus neutralization assays and observed cross-neutralization of SARS-CoV and SARS-CoV-2 with several candidates (Fig. 2A, Table S4). In particular, 8 of the candidates, including 10B8 and 1E4, showed potent neutralization for both SARS-CoV and SARS-CoV-2. Neutralization was observed with all RBD-binding and some NTD-binding antibodies. None of the S2-binding antibodies showed neutralization in SARS-CoV, SARS-CoV-2, or MERS pseudovirus assays (Table S4). Importantly, potent neutralization capability remained for the SARS-CoV-2 variants Alpha, Beta and Delta. While potency was reduced against the Omicron variant for some candidates, it remained high for several candidates—particularly 10B8 (Table S4).

Next, neutralization potency of select candidates were evaluated in SARS-CoV and SARS-CoV-2 authentic virus assays. Overall neutralization potency remained similar to what was observed in pseudovirus assays with two of the potent candidates 10B8 and 1E4 showing IC50 values at sub-nM levels for both SARS-CoV and SARS-CoV-2 (Fig. 2B and C). The bivalent format of 10B8 showed IC50 values of 40 pM and 300 pM for SARS-CoV and SARS-CoV-2, respectively. Even in the monovalent format, IC50 values of 10B8 remained potent (150 pM for SARS-CoV and 2.9 nM for SARS-CoV-2). While the bivalent format of 1E4 remained potent (IC50 values of 50 pM and 200 pM for SARS-CoV and SARS-CoV-2 respectively), the monovalent format of this construct was less potent and IC50 values could not be calculated with the concentrations used in the experiment. Consistent with the literature^14^, REGN10987 Ab, used as a comparator, showed potent neutralization for SARS-CoV-2 but not for SARS-CoV.

### Epitope mapping and structural analysis

We used an Octet-based binning assay to evaluate the epitopes of candidate VHHs. Briefly, the first VHH was incubated with the CoV-2 spike protein, which was captured on the biosensor tip, followed by binding of a second antibody. Benchmark antibodies against the RBD (Class 1: REGN10933^14^, Class 3: REGN10987^14^, and Class 4: VHH-72^15^) were included to classify the RBD-targeting VHHs we discovered^11^. Strikingly, fourteen out of 15 RBD binders binned with VHH-72^15^ known to bind a cryptic cross-reactive Class 4 epitope on the RBD (Fig. S1A)^11^. In contrast, one RBD binder (7A9) did not bin with any of the other candidates or benchmark antibodies tested, indicating a novel epitope. We did not find any cross-reactive RBD-targeting VHHs that bound to either the Class 1 or Class 2 epitopes, consistent with literature that epitopes on the top of the RBD are more variable and strain-specific. Likewise, both NTD binding VHHs (19B8 and 16H7) binned together (Fig. S1B). Regarding S2 binders, three of the VHHs that did not bind to MERS binned together, while one triple cross-reactive VHH (6A1) binned separately from the four double cross-reactive VHHs, indicating that they indeed target distinct epitopes on the S2 domain (Fig. S1B). The other two triple cross-reactive VHHs (S3_29, S3_44) were not evaluated in this assay since they bound well to cell surface expressed spike, but poorly to recombinant proteins (Tables S1–S3).

**Figure 3:**
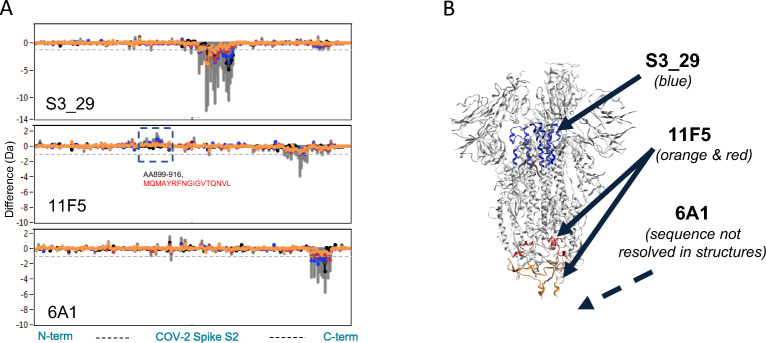
Identification of VHH binding epitopes to guide linker length design. The epitopes of select S2 binders were determined by HDX-MS using the spike protein S2 domain. (**A**) Hydrogen–deuterium exchange difference plots are shown for S3_29, 11F5 and 6A1. Sequence regions showing significant differences are AA962-1006, LVKQLSSNFGAISSVLNDILSRLDKVEAEVQIDRLITGRLQSLQT for S3_29; AA899-916, MQMAYRFNGIGVTQNVL and AA1111-1145, EPQIITTDNFVSGNCDVVIGIVNNTVYDPLQPEL for 11F5; AA1176-1178, VVN and AA1183-1197, DRLNEVAKNLNESL for 6A1. (**B**) The structure of the SARS-CoV-2 Spike (S) protein is shown (PDBID: 6XKL) colored gray. The mapping for S3_29 is shown in blue. The mapping for 11F5 in shown in orange and red marking the sequences protected and deprotected by the VHH respectively. As the region that 6A1 targets has not been resolved in a full spike ectodomain protein structure, it is not shown in this figure.

Unlike the well documented cross-reactive antibodies targeting the RBD, benchmark S2-binding antibodies with known epitopes are scarce. Therefore, we utilized hydrogen–deuterium exchange mass spectrometry (HDX-MS) to map the epitopes of the three classes of S2 binders we identified. These included 6A1 (triple cross-reactive), 11F5 (double cross-reactive), and S3_29 which was triple cross-reactive in the FACS binding assay (Fig. 3, Fig. S2). Our HDX data revealed three distinct binding epitopes for these S2-targeting VHHs with high confidence. The first epitope (shown in blue in Fig. 3B) is located at the top of the S2 domain and targeted by the triple cross-reactive VHH S3_29. This epitope is similar to that of the recently reported MERS cross-reactive antibody 3A3 and includes the S-2P stabilizing mutations that stabilize our spike construct^16^. Given the inability of S3_29 to neutralize wild-type virus and its poor binding to recombinant protein, it is likely that these residues are important for stabilizing the conformational epitope at this dynamic region of the protein rather than being directly important for recognition. The second S2 epitope is located at the stem-helix region at the base of the spike protein and targeted by the triple cross-reactive VHH 6A1. The final epitope is also located at the base of the spike protein but above the stem helix region (shown in orange and red), targeted by the double cross-reactive VHH 11F5 and likely by a few other VHHs that bin together with it (Fig. S1B).

### VHH 7A9 binds a rare RBD epitope and destabilizes the spike trimer

**Figure 4:**
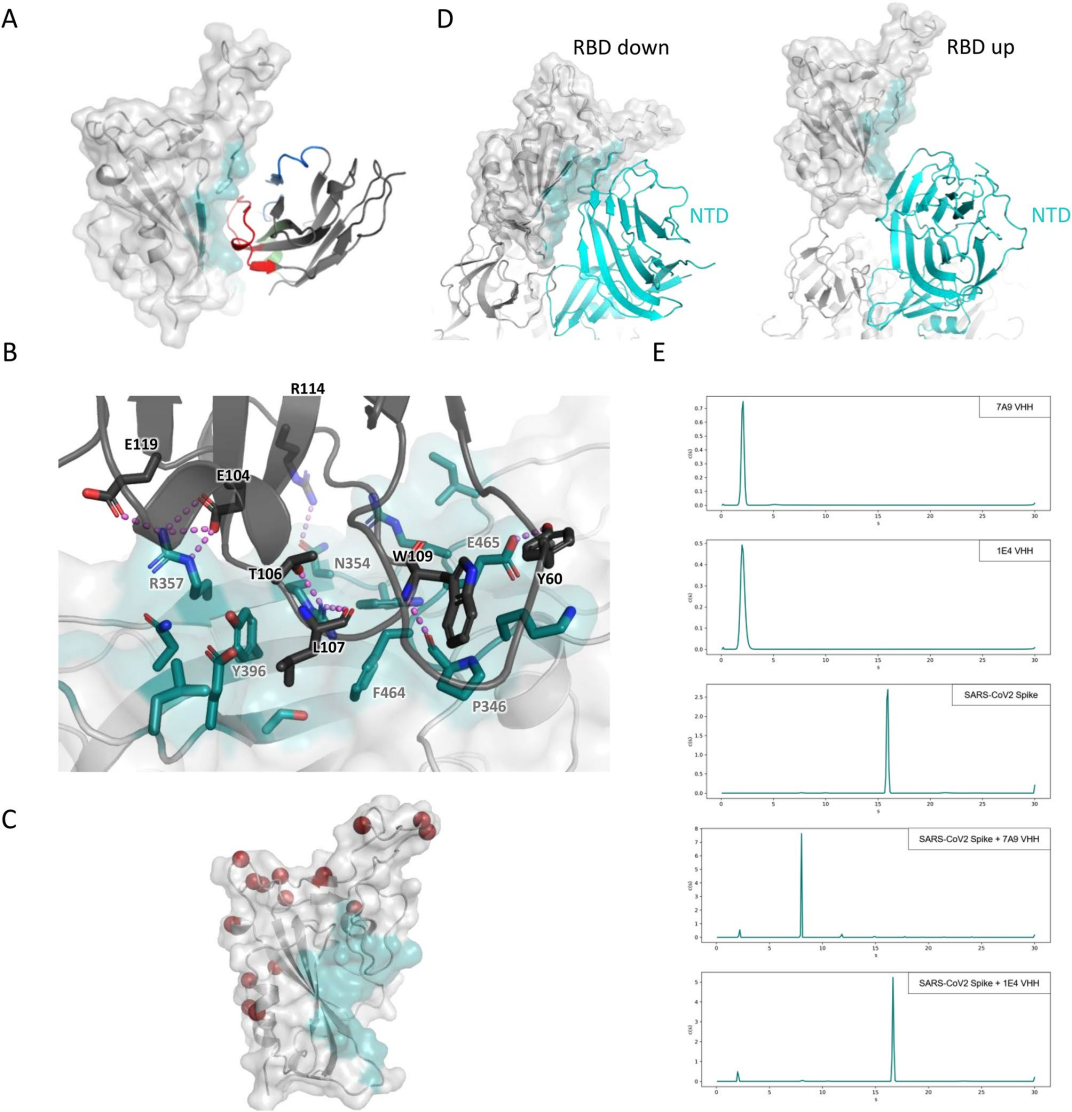
VHH 7A9 binds a rare RBD epitope and triggers spike trimer dissociation. (**A**) Crystal structure of VHH 7A9 bound to the SARS-CoV-2 RBD. The RBD is shown in light grey with the epitope highlighted in teal. The VHH is shown in dark grey with CDR1, CDR2, and CDR3 highlighted in green, blue, and red, respectively. (**B**) Molecular interactions between VHH 7A9 and the SARS-CoV-2 RBD. The RBD is shown in light grey with the epitope residues shown in teal. 7A9 is shown in dark grey. Polar interactions are denoted by violet dashed lines. (**C**) SARS-CoV-2 RBD in cartoon and transparent surface notation with the Cα carbons of residues mutated in the Omicron variant shown as red spheres. The epitope of VHH 7A9 is shown in teal. (**D**) Overlay of the SARS-CoV-2 RBD from the crystal structure onto the closed (left) or open (right) spike protein structures (PDBs: 7DF3 and 6XKL, respectively). (**E**) Continuous distribution (c(s)) analyses of analytical ultracentrifugation data. From top to bottom: 7A9 VHH alone, 1E4 VHH alone, SARS-CoV-2 Spike alone, SARS-CoV-2 Spike + 7A9 VHH, and SARS-CoV-2 Spike + 1E4 VHH.

We were intrigued that the RBD-binding VHH 7A9 bound to a cross-reactive epitope that did not bin with any of the benchmark antibodies used in this study. To characterize the 7A9 epitope, we again used HDX-MS, which indicated that the major binding site of 7A9 is partially occluded by the NTD and spans residues 353–364 of the spike protein (Fig. S3). To further analyze this rare antigenic site, we used X-ray crystallography to determine the structure of 7A9 bound to the CoV-2 RBD (Fig. 4, Table S5). The structure reveals that the third complementarity-determining region (CDR3) of 7A9 forms a platform through which the VHH binds to a concave cleft on the RBD, inserting Leu107 into a small cleft formed by Tyr396 and Phe464. CDR1 and CDR2 contribute only minimally to the interaction, with Arg31 of CDR1 forming a network of Van der Waals interactions and Tyr60 of CDR2 forming a hydrogen bond with Glu465. The energetic driver of the interaction is likely the salt bridges formed between Arg357 of the RBD and two 7A9 glutamates in CDR3, Glu104 and Glu119. A detailed view of the molecular interactions is shown in Fig. 4B. The structure helps to rationalize how 7A9 is still able to bind and neutralize the Omicron variant (Tables S1, S4), as the epitope does not overlap with any of the 15 RBD mutations in this VOC (Fig. 4C). Notably, the 7A9 epitope is fully occluded in the closed state and partially occluded in the open state of the spike protein (Fig. 4D). Modeling 7A9 binding onto the full CoV-2 spike protein indicates that the VHH would introduce a clash with the NTD from the neighboring spike protomer in either the closed- or open state (Fig. S4). This suggests that either a conformational shift in the RBD relative to the NTD or trimer dissociation would have to occur upon VHH binding. Indeed, recent HDX studies and advances in analysis of heterogeneity in cryo-EM data have shown that the spike protein is quite dynamic and samples states that would potentially expose the 7A9 epitope^17,18^.

To understand the structural consequences of 7A9 binding to the SARS-CoV-2 spike trimer, we conducted a series of analytical ultracentrifugation sedimentation velocity (AUC-SV) experiments, using a continuous distribution (c(s)) analysis to determine the species present in solution (Fig. 4E, Fig. S5A). The c(s) distribution of the spike alone showed as a single peak (95% of total signal) at 15.9 s, corresponding to an apparent mass of 441 kDa, consistent with a stable trimer (estimated mass of 398 kDa). 7A9 VHH showed as a single peak (93% of total signal) at 2.1 s, corresponding to an apparent mass of 18 kDa, as expected. 7A9 was then mixed with the spike trimer at a 1.5:1 (monomer:monomer) molar ratio. Compared to the spike trimer alone, this mixture displayed a dramatic shift in the sedimentation profile that is readily apparent from the absorbance scans (Fig. S5A). The c(s) distribution revealed 2 major species at 2.2 s (7%) and 8.0 s (86%), with apparent masses of 22 and 155 kDa, respectively. We interpret these species as excess free 7A9 VHH and 7A9 bound to a spike monomer (theoretical mass of 151 kDa). Strikingly, there was no appreciable intact spike trimer (15.9 s) remaining after mixing with 7A9, indicating that binding of 7A9 completely dissociated the spike trimer. To determine if spike trimer dissociation was specifically due to 7A9 binding, we ran identical AUC-SV experiments using the 1E4 VHH, which is predicted to bind to, but not disrupt, the spike trimer. Similar to 7A9 alone, the c(s) distribution of 1E4 alone showed a single peak (98% of total signal) at 2.1 s, corresponding to an apparent mass of 19 kDa. Mixing 1E4 with the spike trimer at a 1.5:1 (monomer:monomer) ratio showed 2 major species at 2.0 s (8%) and 16.7 s (88%), with apparent masses of 20 and 467 kDa, respectively. We interpret these species as excess free 1E4 VHH and 1E4 bound to the spike timer (theoretical mass of 451 kDa). Further, we analyzed identical samples using size-exclusion chromatography coupled to multi-angle light scattering (SEC-MALS) and performed molecular weight calculations via a conjugate analysis method (Fig. S5B). Consistent with the AUC-SV experiments, we observed dissociation of the spike trimer upon binding to 7A9, but not 1E4, with calculated masses of the complexes being comparable to AUC-SV experiments (Fig. S5C). Together, these data clearly indicate that the binding of 7A9 to its cryptic epitope results in the complete dissociation of the SARS-CoV-2 spike trimer, whereas the spike trimer remains intact upon 1E4 binding.

### Multimeric design enhances neutralization

It has been reported that multimerization of VHHs can lead to improvement in potency^8,19,20^, and our data described earlier in this manuscript (Fig. 1B) suggested that bivalent VHHs indeed have improved antigen binding affinity compared to their monovalent counterparts. Given both the size of the spike protein as well as its trimeric nature, we employed two approaches to achieve higher potency and potentially protection against escape mutations. The first approach was to design homotrimers of RBD-binding VHHs (1E4, 7A9 and 10B8) to engage each monomer of the spike protein simultaneously and improve potency. Alternatively, we also designed heterotrimers targeting three different sites on the spike protein, which would in theory be more resistant to escape mutants, as the loss of affinity at any one site could be compensated by the binding at the other sites. A number of homo- and hetero-trimeric constructs were designed using the results of domain binding, epitope mapping, and structural modeling to determine the optimal lengths of the linkers between each VHH.

**Figure 5:**
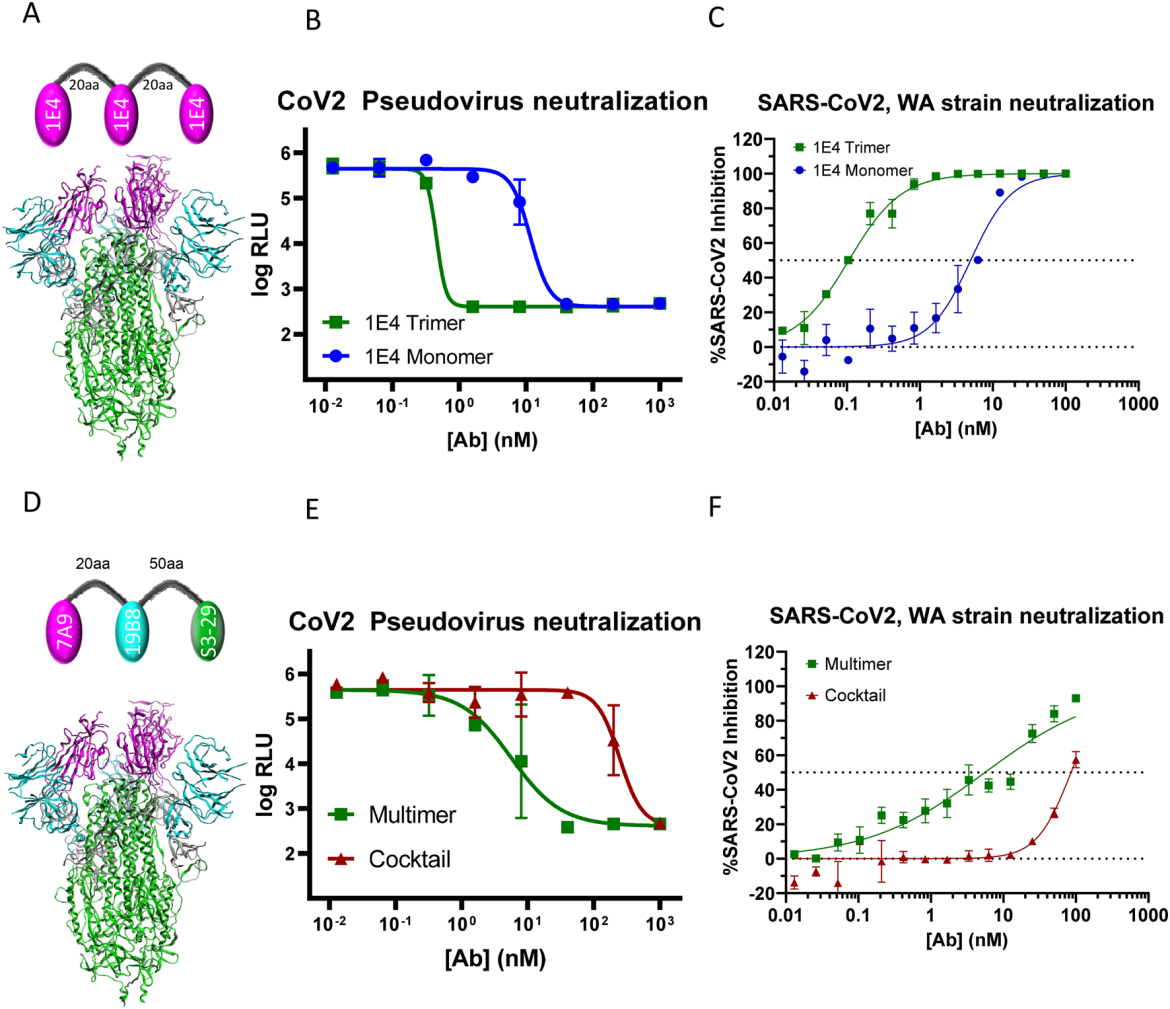
Multimerization of VHH results in enhanced neutralization potency. (**A**) Design of homotrimeric VHH multimer with 20 aa linker (4 × GGGGS) in between monomeric VHH 1E4. The structure of the SARS-CoV-2 Spike (S) protein (PDBID: 6XKL) is colored by domain as in Fig. 1. (**B**) SARS-CoV-2 VSV pseudovirus neutralization by 1E4 VHH monomer vs 1E4 VHH trimer. (**C**) SARS-CoV-2 authentic virus neutralization by 1E4 VHH monomer vs 1E4 VHH trimer. (**D**) Design of heterotrimeric VHH multimer with 20 aa linker (4 × GGGGS) in between VHH 7A9 and VHH 19B8 and 50 aa linker (GAS) in between VHH 19B8 and VHH S3_29. The structure of the SARS-CoV-2 Spike (S) protein (PDBID: 6XKL) is colored by domain as in Fig. 1. Heterotrimeric design with 20 aa linker (4 × GGGGS) in between VHH 7A9 and VHH 19B8 and 50 aa linker (GAS) in between VHH 19B8 and VHH S3_29. (**E**) SARS-CoV-2 VSV pseudovirus neutralization by 7A9-19B8-S3_29 multimer vs combination of 7A9, 19B8 and S3_29 in equimolar concentrations. (**F**) SARS-CoV-2 authentic virus neutralization by 7A9-19B8-S3_29 multimer vs combination of 7A9, 19B8 and S3_29 in equimolar concentrations.

To determine whether multimerization enhances potency, we compared neutralization potency of multimeric constructs with cocktail mixtures of individual VHHs used in the respective multimer (Fig. 5, Table S6). A homotrimer of 1E4 showed about a 26-fold improvement in neutralization of SARS-CoV-2 pseudovirus and about 46-fold improvement in neutralization of SARS-CoV-2 authentic virus (Fig. 5A–C). Likewise, a heterotrimeric construct targeting RBD, NTD and S2 showed a 43-fold improvement in neutralization over a cocktail of the same three VHHs in SARS-CoV-2 pseudovirus assay (Fig. 5D,E). Importantly, this potency improvement was also observed in authentic SARS-CoV-2 neutralization (Fig. 5F). Additionally, we observed multimerization leading to improved potency with several designs where weakly-neutralizing or non-neutralizing VHHs could be combined to enhance potency (Table S6).

## Discussion

In this study, we discovered a variety of cross-reactive VHHs against the coronavirus spike protein and characterized the binding affinity, neutralization potency and the epitopes these VHHs target. While the affinities of these VHHs in their monomeric format ranged from < 1 nM to > 100 nM, affinities < 1 nM were observed for the bivalent VHH-Fc format. We found that some of the VHHs have exceptional binding affinity and neutralization potency against SARS-CoV-2 that are comparable to the benchmark antibodies being used in the clinic or in clinical development (Fig. 2C). In addition, these potent antibodies can also neutralize the related coronavirus SARS-CoV and retain activity against the newly emerged Omicron VOC.

The cross-reactive VHHs we identified bind distinct epitopes on the spike protein. The majority of the antibodies cross-react to SARS-CoV and SARS-CoV-2, but not to MERS, which was expected as SARS-CoV and SARS-CoV-2 are more related and both belong to the Sarbecovirus subfamily. Most of the VHHs we identified target the RBD domain, and all of the RBD-targeting VHHs are neutralizing. This finding is consistent with literature where at least two distinct cross-neutralizing epitopes have been identified on the RBD (Classes 3 and 4)^11^. Interestingly, most of the RBD VHH antibodies we found target the cryptic Class 4 epitope (targeted by VHH-72^15^ and CR3022^13^), suggesting that the smaller size of VHH might indeed facilitate interaction with this less accessible antigenic site.

Among the cross-reactive RBD targeting VHHs we discovered, 7A9 is unique, as it is the only VHH identified in this study that is both cross-reactive and does not clearly bin with any other of the Class 1–4 epitopes. Our combined structural approaches clearly demonstrate that 7A9 binds to a highly conserved and cryptic epitope on the RBD that is partially occluded by the CoV-2 spike NTD, providing a structural basis for the retained binding and neutralization of 7A9 to the Omicron variant. Importantly, our data also demonstrates that 7A9 binding leads to the dissociation of the CoV-2 spike trimer. Recently, there have been two reported structures of antibodies that bind to a similar cryptic RBD epitope: the bn03 bispecific VHH (n3130v segment) and 553–49 IgG^21,22^. Similar to 7A9, both of these antibodies bind to sites that are partially occluded by the NTD of a neighboring protomer in the CoV-2 spike trimer and induce dissociation of the trimer upon binding. However, comparison of the structures of 7A9, bn03, and 553–49 show unique modes of binding for each (Fig. S6A). Comparison of the footprints of each antibody show that the epitopes of 553–49 and bn03 share very little overlap with one-another. The binding site of 7A9 is located in between that of 553–49 and bn03 and shares epitope residues with each of these antibodies (Fig. S6B and C). The divergent mechanisms of these three antibodies are further highlighted by the fact that only RBD residues R355 and R357 are located near the interfaces of all three (Fig. S6C and D). Therefore, each of these antibodies can achieve broad specificity and induce trimer dissociation via distinct mechanisms of binding. It is unclear why these agents exhibit differing levels of viral neutralization, which could be the result of multiple factors including size, binding geometry and valency. Interestingly, several antibodies have been reported in recent years that dissociate other trimeric viral fusion proteins including influenza hemagglutinin and respiratory syncytial virus fusion protein, suggesting that this might a common mechanism of antibody neutralization against respiratory viruses^23,24^.

A few of the VHHs that were cross-reactive to both SARS-CoV and SARS-CoV-2 target NTD and S2, and while some of the NTD binders are neutralizing, none of the S2 binders neutralize. We also found three highly cross-reactive VHHs that also bound to spike protein from MERS, which belongs to a separate subfamily from CoV and CoV-2. All these three VHHs target the S2 domain but did not neutralize any of the three viruses. These data suggest that S2 domain is more conserved and presents multiple distinct cross-reactive epitopes. While these cross-binding S2 antibodies are non-neutralizing, it is possible that they can confer in vivo protection through other Fc-mediated mechanisms. We therefore performed additional characterization to understand the specific epitopes they target. From our HDX mapping data, we observed three distinct binding epitopes for S2-targeting VHHs with high confidence. The first epitope is represented by S3_29, which share sequence similarity with one other VHH, and targets the top of the S2 domain. In the closed state of the Spike protein, this region is not exposed or accessible to VHHs. This region could be exposed for VHH binding in one of two ways: (a) when multiple RBDs are in the open state, or (b) following shedding of S1 but prior to the conformational change that precedes membrane fusion. Intriguingly, there is a previous report of an antibody that targets the same region that was mapped similarly using HDX, providing further evidence that this site is accessible^25^. The second epitope is represented by 6A1 which targets the stem helix region at the base of the spike protein. While not resolved in most spike protein structures, previous studies have reported similar antibodies^26–29^. This region is exposed regardless of the conformational state of the prefusion Spike protein. Our binding data suggest that this epitope is the broadest from this study, with reactivity not only to Sarbecovirus and MERS, but also to endemic human coronaviruses HKU1 and OC43 which belong to a different beta coronavirus subfamily from CoV/CoV-2 and MERS. Our data suggests that this region of the spike protein is likely the most conserved. In the future, it would be interesting to evaluate whether antibody binding to this epitope can offer protection against coronavirus infection and disease. The final S2 epitope is represented by 11F5, which binds to the base of the spike protein. Interestingly, this antibody appears to cause both protection and deprotection effects upon binding to the bottom of the spike in our HDX experiment, suggesting that spike undergoes a local conformational change when this antibody binds.

Most of our VHH multimer designs led to potency enhancement, while two designs showed modest loss of potency over their respective VHH cocktails. Significant enhancement in potency was observed with several of our multimer designs. One of the homotrimeric designs, 1E4, showed significant potency enhancement over its monomeric version. However, homotrimers of 10B8 and 7A9 showed no potency enhancement over their respective monomers. One potential reason for this could be due to lack of simultaneous binding to all three protomers with the current design. While homotrimeric VHH constructs targeting RBD epitopes have been shown to enhance potency compared to their monomeric counterparts^8,19,20^, our study also showed significant potency improvement with heterotrimeric constructs compared to their respective cocktail mixtures. In particular, we observed significant enhancement in potency when heterotrimers were engineered using either weakly-neutralizing or non-neutralizing VHHs. In contrast, when the multimers were designed using strong neutralizers, potency enhancements observed over respective VHH cocktails were modest. Interestingly, most of the multimeric VHHs that showed the highest fold increase in neutralization potency appear to include the S3_29 VHH. We speculate that this epitope, which is located relatively closer to the S1 domain of spike, might help to stabilize the RBD or NTD targeting components to bind their epitopes. It is possible that simultaneous engagement of multiple epitopes constrains the conformation of the spike protein, further resulting in potency enhancement. This approach also has the potential to protect against escape mutations and could be valuable in addressing the loss of potency observed with several clinical candidates with the emergence of SARS-CoV-2 variants. A similar strategy could be applied to development of therapeutics against other pathogens that display significant divergence.

## Materials and methods

### Plasmid construction, protein expression and protein purification

#### Spike protein

The SARS-CoV prefusion-stabilized spike protein (SARS-CoV-PreS) includes the SARS-CoV spike protein ectodomain residues 1–1190 (amino acid 1 denotes the starting methionine in the signal peptide) two proline substitutions at K968P and V969P, a C-terminal T4 fibritin trimerization domain, an HRV3C protease cleavage site, and an 8 × His-tag as described previously^30^. The SARS-CoV spike ectodomain protein where the His-tag was not cleaved (CoV-PreS-3C) contained a thrombin cleavage site in place of the HRV3C protease site. The MERS prefusion-stabilized spike protein (MERS-PreS) includes the MERS spike protein ectodomain residues 1–1291, two proline substitutions at V1060P and L1061P, an “ASVG” substitution at the furin cleavage site (residues 748–751, RSVR), a C-terminal T4 fibritin trimerization domain, a thrombin cleavage site, and an 8 × His-tag similar to previously described^31^. The SARS-CoV-2 prefusion-stabilized spike protein (SARS-CoV-2-PreS) includes SARS-CoV-2 spike protein ectodomain residues 1–1208, two proline substitutions at K986P and V987P, a “GSAS” substitution at the furin cleavage site (residues 682–685, RRAR), a C-terminal T4 fibritin trimerization domain, a thrombin cleavage site, and an 8 × His-tag similar to previously described^32^. The “closed” conformation SARS-CoV-2 spike protein trimer (CoV-2-PreS-Closed) contains the SARS-CoV-2 spike protein ectodomain residues 1–1208, four amino acid substitutions (D614N, A892P, A942P,and V987P), a thrombin cleavage site, and an 8 × His-tag similar to previously described^33^. The Receptor Binding Domain (RBD) and N-Terminal Domain (NTD) protein constructs were designed as follows. The CoV-2-S-RBD-SD1 spike and CoV-2-S-NTD spike constructs contain residues 319–591 and 1–305 respectively cloned in frame with the native CoV-2 signal sequence and appended with a C-terminal T4 fibritin trimerization domain, a thrombin cleavage site, and an 8 × His-tag similar to previously described^32^. The CoV-S-RBD-SD1 and MERS-S-RBD-SD1 spike protein constructs contain residues 306–577 and 367–655 respectively cloned in frame with the native protein’s signal peptide, a thrombin cleavage site, and an 8 × His-tag. The S2 domain only SARS-CoV-2 spike protein (CoV-2-PreS-S2) construct includes ectodomain residues 697–1208 with two proline substitutions at K968 and V969 cloned in frame immediately downstream of an IgK signal peptide and appended with a T4 fibritin trimerization domain, thrombin cleavage site and 8 × His-tag at the C-terminus. Biotinylated constructs include an Avi-tag sequence immediately upstream of the protease cleavage site flanked by GRS—GG linkers. The CoV-2-S-RBD construct used for x-ray crystallography contains residues 319–541 and was cloned in frame with the native CoV-2 signal sequence and contained a C-terminal SG-6xHis. All gene-encoding regions were mammalian codon-optimized and subcloned into a eukaryotic-expression vector under the control of the CMV promotor.

Plasmids were transiently transfected into Expi 293F cells (ThermoFisher) using Expifectamine (ThermoFisher) following manufacturer’s recommended protocol. Cell supernatants were harvested 72 h post-transfection and clarified by centrifugation at 10,700 × *g* at 20 °C for 30 min. PS-20 was added at a final concentration of 0.01% to the clarified supernatants to mitigate aggregation. Clarified supernatants were aliquoted into 250 mL Corning bottles and transferred to − 70 °C for storage until purification.

Clarified supernatant was thawed in a 37 °C shaking water bath and carried forward into purification. Spike protein was purified using immobilized metal affinity chromatography (IMAC) on a HisTrap Ni Sepharose Hi Performance column (Cytiva) on a fast protein liquid chromatography system. The purification tag was cleaved with either thrombin (Cytiva) or HRV3C (Accelagen) protease and was incubated overnight at 25 °C or 4 °C, respectively, with gentle stir to remove the imidazole. For proteins with similar molecular weight to thrombin, the His-tag from the recombinant proteins was cleaved using the Thrombin CleanCleave™ Kit (Sigma-Aldrich) following manufacturer’s recommended protocol.

Spike protein was further purified by a second, subtractive IMAC step on a HisTrap FF column (Cytiva) to separate cleaved Spike proteins from protease, contaminants, and uncleaved Spike. If biotinylation is required, purified Spike protein was biotinylated after subtractive IMAC using Biotinylation Kit (Avidity LLC) according to the manufacturer’s recommended protocol. Final purification step of the sample was achieved with size exclusion chromatography (SEC) using a HiLoad™ 26/600 Superdex™ 200 pg column (Cytiva) for SARS-CoV-2-PreS and MERS-PreS and a HiPrep™ 26/60 Sephacryl S-300 HR column (Cytiva) for SARS-CoV-PreS with 50 mM HEPES, pH 7.5, 300 mM NaCl (150 mM NaCl for SARS-CoV-2-PreS-Closed) running buffer. Fractions containing protein of interest were pooled based on SEC chromatograms, concentrated, if required, and filtered prior to aliquoting and flash-freezing in liquid nitrogen. Final protein concentration was quantified using NanoDrop™ 2000c Spectrophotometer (Thermo Scientific) A_280_ analysis after the small amount of sample was denatured using 6 M guanidine hydrochloride. Protein purity was estimated by the intensity of the bands on a Coomassie-stained gel. Protein oligomerization state and size were confirmed using size-exclusion chromatography multiangle laser light scattering (SEC–MALLS).

Construct design, expression and purification of human MPV post-fusion F trimer has been described previously^34^.

The construct used for x-ray crystallography, SARS-CoV-2-S-RBD (519–541) was harvested 72 h post-transfection, supernatant was clarified by centrifugation, and concentrated/buffer exchanged by tangential flow filtration into 25 mM HEPES pH 7.5, 300 mM NaCl. Sample was purified by IMAC over a HisTRAP FF column (Cytiva) and subsequent Superdex 75 column in 25 mM HEPES pH 7.5, 150 mM NaCl. Sample was concentrated to 10 mg/mL and flash frozen. The 7A9 VHH was harvested 6 days post-transfection and clarified. Supernatant was purified by IMAC over a HisTRAP Excel column (Cytiva) in 25 mM HEPES pH 7.5, 300 mM NaCl and subsequent Superdex 75 in 25 mM HEPES pH 7.5, 150 mM NaCl. Sample was concentrated to 10 mg/mL and flash frozen. For the RBD/7A9 VHH complex, samples were thawed and mixed using excess VHH 1:1.5 (RBD:VHH) and incubated at 4C for one hour. Complex was then purified over a Superdex 75 column in 25 mM HEPES pH 7.5, 150 mM NaCl. Final purified complex was concentrated to 10.7 mg/mL and flash frozen.

Recombinant SARS-CoV-2 spike proteins from different variants used in ELISA were obtained from Acro Biosystems—B.1.1.7 variant (Cat# SPN-C52H6), B.1.351 variant (Cat# SPN-C52Hk), P.1 variant (Cat# SPN-C52Hg), B.1.617.2 variant (Cat# SPN-C52He), BA.2 (Cat# SPN-C5223), BA1.1 (Cat# SPN-C52Hz).

#### VHH monomer, VHH-Fc and conventional antibodies

VHH monomer, VHH-Fc, and human IgG1 heavy (HC) and light (LC) antibody chains were separately cloned into our in-house pTT5 based vector carrying Lonza leader secretion tags and CMV promoter. VHH-Fc and VHH monomer plasmids were transiently transfected into ExpiCHO expression system using serum-free defined media, and suspension adapted CHO cells following manufacturer recommendations with Max-Titer protocol. Plasmids for IgG1 conventional antibody HC and LC were transiently transfected at 1:1 ratio into ExpiCHO expression system using serum-free defined media following manufacturer recommendations with Max-Titer protocol. Cells were harvested after 7-days with feeds on day 1 and 5 and temperature shift to 32C on Day1. High-throughput (HT) Protein A (Mabselect) affinity chromatography in miniature columns (Robocolumns) was used for capture and enrichment of recombinant antibodies from clarified harvest cell culture fluid (HCCF). Analytical size-exclusion (aSEC) to characterize solution behavior and capillary electrophoresis (CE-SDS) to characterize under denaturating conditions was used to QC the expressed molecules. An acceptable criteria of overall purity was chosen for selected molecules and the antibodies were typically > 90% purity as determined by aSEC.

#### VHH-multimer

VHH multimer antibody chains were generated by fusing 3 VHHs together with different linker lengths and cloning into our in-house pTT5 based vector. Linkers used were either 20 aa (4 × GGGGS), 50aa (5 × GSAGSAAGSG)^35^, or 90aa (4.5 × ASPAAPAPASPAAPAPSAPA)^36^. VHH multimer plasmids were transiently transfected into Expi293 expression system in suspension using serum-free defined media following manufacturer recommendations. Cell culture supernatants were harvested after 5 days with feed on day 1. Ni affinity (Ni Sepharose Excel – Cytiva) chromatography using gravity flow columns enabled 1-step purification of recombinant antibodies from clarified harvest cell culture fluid. aSEC was used to characterize the multimers.

### Llama immunization

All animal procedures described in this study were approved by the Institutional Animal Care and Use Committee of Capralogics, Inc., a USDA regulated research facility. All animal work performed were in compliance with the Guide for the Care and Use of Laboratory Animals published by the National Research Council^37^ the American Veterinary Medical Association Guidelines for the Euthanasia of Animals^38^, and the ARRIVE guidelines^39^.

Four llamas were immunized at Capralogics using a heterologous recombinant protein immunization boost strategy. This approach involved an initial subcutaneous injection of all four llamas with 0.5 mg of SARS-CoV-2 spike protein mixed with Complete Freund’s adjuvant. For the remaining two injections, two llamas received incomplete Freund’s adjuvant (IFA) and two llamas received no additional adjuvant. The injections were all three weeks apart and included a second subcutaneous injection of 0.5 mg of MERS protein and then a third subcutaneous injection of 0.5 mg of SARS-CoV spike protein. Ten days after the final injection, 200 mL of whole blood was collected from the llamas in heparin blood collection tubes. Peripheral Blood Mononuclear Cells (PBMCs) were then isolated from the blood using density gradient centrifugation. Serum titers were also evaluated against SARS-CoV-2, SARS-CoV, and MERS spike protein by ELISA and two llamas were selected for further processing (one that used an IFA injection strategy and one that used no adjuvant for injections other than the primary injection.)

### Llama B-cell sorting, culture, and sequencing

Purified llama PBMCs from the immunized animals were cell surface stained using biotinylated SARS CoV-2 or biotinylated MERS spike protein, goat anti-llama IgG (H + L) FITC conjugate (Thermo Cat# A16061), 7-AAD live/dead cell stain (Biolegend, Cat# 420404), and streptavidin BV421 (Biolegend Cat# 405226). Live B-cells, positive for biotinylated antigen and IgG, were sorted as single cells into 96-well plates or sorted in bulk into tubes using a BD FACSAria Fusion.

The single sorted cells were cultured for two weeks with a gamma irradiated CD40L-EL4 recombinant cell line and internally made llama IL-2/IL-21 cytokines at 37C. After two weeks, B-cell culture supernatants were screened using ELISA and FACS for binding to recombinant or cell expressed proteins. Candidates of interest were then lysed in 85 uL of Qiagen TCL buffer with 1% B-mercaptoethanol and RNA was isolated using Qiagen Turbocapture tubes (Qiagen Cat# 72251).

cDNA was generated using SuperScript IV reverse transcriptase (Thermo, Cat #18090050) in the presence of a template switching oligo (TSO) for 5’RACE. A first round PCR reaction was then performed using GoTaq polymerase (Promega, Cat # M7422), a llama heavy chain only gene specific constant region reverse primer, and a TSO-compatible forward primer. A second round of PCR, also using GoTaq polymerase, further amplified the PCR products and introduced Illumina MiSeq NGS adaptors and indices for high-throughput, multiplexed NGS sequencing. Individual, indexed PCR products were then pooled and gel purified using a Qiagen gel purification kit (Qiagen Cat# 28704.) Sequences that showed cross-reactivity between 2 or more strains by ELISA or FACS were identified and recombinantly expressed for further testing.

The bulk sorted B-cells were immediately lysed in Qiagen buffer RLT with 1% B-mercaptoethanol, and RNA was isolated using the Qiagen RNeasy micro kit (Cat# 74004.) cDNA was generated using Superscript IV reverse transcriptase (Thermo, Cat #18090050) in the presence of a template switching oligo (TSO) containing a unique molecular identifier (UMI) to enable 5’RACE and error correction during analysis of the NGS sequencing data. Two rounds of PCR were then performed using KAPA HiFi Polymerase (KAPA, Cat # KK2501), a llama heavy chain only gene specific constant region reverse primer, and a TSO-compatible forward primer. The primers also included indices to identify specific samples and Illumina MiSeq adaptors. Individual, indexed PCR products were then pooled and gel purified using a Qiagen gel purification kit (Qiagen Cat# 28704.) Bioinformatic analysis was used to compare sequencing data from B-cells sorted using SARS CoV-2 spike protein and MERS spike protein to look for sequences in common between the two libraries, indicating potential cross-reactivity of a sequence for at least two strains. Two candidates identified using this approach were recombinantly expressed.

### Protein ELISA

96-well half area plates were coated with either 25 uL/well of full-length spike proteins or domain proteins (1 µg/mL in PBS buffer) and incubated overnight at 4 °C. The next day, plates were washed 3 times with PBST (PBS + 0.05% Tween 20) and blocked with 25 uL/well of blocking buffer (PBS with 5% FBS) for 30 min at room temperature. B-cell culture supernatant or titrated purified antibody was then transferred at 25 ul/well to the 96-well plates and incubated for 60 min at room temperature. The plates were then washed 3 times with PBST. Then 25ul/well of rabbit anti-llama IgG (H + L) HRP (1:1000 dilution in blocking buffer, Thermo Scientific Cat# A16154) or a secondary antibody specific for the Fc of the purified antibody was added to the plates and incubated for 60 min at room temperature. Finally, the plates were washed 5 times with PBST and developed by adding TMB reagent (Thermo Cat# 34,029) to the plates for 2–3 min. The reactions were stopped with 0.16 M sulfuric acid and the absorbance read at 450 nm and 650 nm using a spectrophometer. EC50 values for protein binding were calculated using the non-linear regression curve fitting tool in GraphPad Prism for log(agonist) vs response (three parameters), using optical density (OD) data collected at each concentration in duplicate.

### Multiplex FACS cell binding

Recombinant CHO K1 cells lines stably expressing spike proteins from different virus strains (SARS-CoV, SARS-CoV-2, MERS, HKU1 and OC43) were generated using aa 1–1236 from SARS-CoV, aa 1–1254 from SARS-CoV-2, aa 1–1337 from MERS, aa 1–1334 from HKU1, and aa 1–1336 from OC43^40^. Cell were cultured in a shaking incubator at 37C, 6% CO2, 75% humidity, and 120RPM. The cells were grown in CD CHO media (Gibco, Cat# 12490) with 4 mM L-glutamine (Avantor, Cat# 2078), 1% Hypoxanthine/Thymidine mix (Gibco Cat# 11067), 4 mg/L Blasticidin (Gibco, Cat# A11139) and 200 mg/L zeocin (Invitrogen, Cat# R25005), harvested using trypsin, and washed twice with PBS buffer. Cell trace dye (CellTrace-violet Thermo Scientific Cat# C34557 and CellTrace-Far Red Thermo Scientific Cat# 34564) was diluted at optimized concentrations in PBS (1 ml staining volume/10 M cells) and used to resuspend cell pellets prepared from the different recombinant cell lines. The cells were incubated with the dyes at 37 °C for 30 min in the dark, with occasional swirling. These staining reactions were stopped by adding warm DMEM/F12 complete medium with 10% FBS, using 5X the original staining volume, and incubated at 37 °C for 10 min. Stained cells were spun down and washed once with 10 ml PBS and resuspended in FACS buffer (5% fetal bovine serum in PBS). Cells were then checked to confirm both positive staining with the dyes and that each recombinant cell line demonstrated a separate fluorescent intensity using an Intellicyt (Sartorius). The different recombinant cell lines were then mixed by resuspending in FACS buffer and aliquoted into 96well plates (50ul/well, 80X10^4/well). Cells were stained with either 50 ul of titrated, purified antibodies or B-cell culture supernatant for 30 min and then spun down and washed 1X with FACS buffer. Finally, the cells were stained with a fluorescently labeled secondary antibody (specific to the Fc domain of the supernatant or recombinant antibody) for 30 min and spun down and washed 2X with FACS buffer. Cells resuspended in 50ul FACS buffer were then analyzed using an Intellicyt. EC50 values for FACS binding were calculated using the non-linear regression curve fitting tool in GraphPad Prism for log(agonist) vs response (three parameters), using mean fluorescence intensity (MFI) data collected at each concentration in duplicate.

### Affinity measurement

VHH affinities and VHH-Fc bivalent apparent affinities were measured on a Biacore 4000 (Cytiva). SARS-CoV-2 PreS Spike ECD protein was biotinylated via a C-terminal Avi tag and immobilized at 600 RU to 1500 RU on a streptavidin coated Series S Sensor Chip SA (Cytiva, Cat. No. 29699621). VHHs were injected at 3.7 nM to 300 nM and VHH-Fcs were injected at 2.5 nM to 200 nM. Injections were 3 min long and dissociation was monitored for 10 min. The running buffer was HBS EP + (10 mM HEPES, 150 mM NaCl, 0.05% Tween20, pH 7.4) and 25 °C. The surface was regenerated with a 30 s injection of Glycine pH 1.9. Data were fit to a 1:1 binding model using Biacore Evaluation software version 1.1.

### Octet epitope binning

Tandem binning experiments were performed on Octet-HTX (Sartorius) using BLI technology to bin anti-CoV-2-spike protein antibodies. Biotinylated CoV-2 Spike ECD PreS-AVI protein (20 nM) was captured on a streptavidin (SA) coated biosensors (Sartorius) for 20 min to get 1.5–2 nm binding response. First antibody (20-40ug/mL) was flowed over to the trimer SARS-CoV-2 spike protein until all the binding sites get saturated, followed by the binding of 20–40ug/mL of 2nd antibody. Benchmark Class 1, Class 3 and Class 4 binders were used to classify RBD domain binder. Four in-house generated antibodies were used to screen NTD and S2 domain binders. Kinetic buffer (1XKB, PBS + 0.02% Tween20, 0.1% BSA, 0.05% sodium azide) was used as a running buffer. Biosensors were regenerated (1.7 nM Glycine) followed by a buffer wash twice after each cycle. Black 384 well polypropylene tilted bottom plates were used for binning experiment.

### Generation of recombinant VSVΔG-based pseudoviruses carrying firefly luciferase (Luc) reporter gene and coronavirus spike (S) proteins

Pseudovirus particles containing rVSVΔG-Luc constructs were made as previously described^40–42^. Coronavirus spike proteins CoV-2-SΔ18 WT or variants, SARS-SΔ19, or MERS-SΔ16 were used in current study.

### rVSV∆G-Luc pseudovirus neutralization assay

Pseudovirus neutralization assay and IC50 calculation were performed as previously described in Ref.^40^ with slight adaptation: Vero E6 cells (ATCC) were used at 22,000 cells/well for MERS in addition to 293 T ACE2 cells used as described for SARS-CoV-2 WT and variants and SARS-CoV.

### Generation of authentic SARS-CoV and SARS-CoV-2

All work with authentic SARS-CoV and SARS-CoV-2 viruses were completed in BSL-3 laboratories at United States Army Medical Research Institute of Infectious Diseases (USAMRIID) in accordance with federal and institutional biosafety standards and regulations as described before^40^.

### Authentic SARS-CoV and SARS-CoV-2 IFA neutralization assay

Authentic SARS-CoV/Urbani, and SARS-CoV-2 neutralization assays were completed at USAMRIID as described before^40^.

### Hydrogen–Deuterium exchange mass spectrometry

To map the binding epitope for S3_29, 11F5 and 6A1, HDX-MS experiments were carried out using a spike protein S2 domain (SARS-COV-2-PreS-S2-His) described above. The protein solution was 1.9 mg/mL (MW ~ 60 kDa, equivalent to ~ 30 µM) in 50 mM HEPES, 300 mM NaCl, and pH 7.5 buffer. To form S2:VHH complex, equal volume of 30 µM S2 was incubated with 30 µM VHH at 4 °C for 30 min as stock solution. HDX-MS experiments were performed using an automated HDX system (Waters Corporation, MA, USA) as previously described^43^. Briefly, six microliters of S2 or S2:VHH stock solution were diluted tenfold with labeling buffer at 20 °C to initiate the deuterium exchange reaction. The labeling time points were 0, 10 s, 1, 10, and 100 min. Fifty microliters of diluted protein solution were mixed with an equal volume of the quench buffer (8 M urea, 1 M TCEP in 100 mM phosphate buffer, pH 2.5) at 4 °C, of which 90 µL was injected to the LC/MS system. Online digestion was performed using an immobilized protease type XIII/pepsin column (w/w, 1:1), 2.1 × 30 mm (NBA2014002, NovaBioAssays, LLC, MA, USA).

To map the binding epitope for 7A9, HDX-MS experiments were performed using SARS- CoV-2 RBD domain described above. The protein solution was 2.6 mg/mL (MW ~ 31 kDa, equivalent to ~ 83.5 µM) in 10 mM sodium phosphate, 75 mM sodium chloride, 3% sucrose, and pH 7.4 buffer. CoV-2 RBD and 7A9 VHH complex was prepared by mixing 15 µL 2.6 mg/mL recombinant CoV-2 RBD with 11.7 µL 3.89 mg/mL 7A9 VHH and 43.3 µL PBS. The recombinant CoV-2 RBD alone was prepared by mixing 15 µL 2.6 mg/mL recombinant CoV-2 RBD with 55 µL PBS. 10 µL of the recombinant CoV-2 RBD alone or RBD:VHH complex was incubated with 90 µL of control buffer (50 mM phosphate, 100 mM sodium chloride at pH 7.4) or deuterium oxide labeling buffer (50 mM sodium phosphate, 100 mM sodium chloride at pD 7.0). The labeling time was 0 s, 15 s, 60 s, 600 s, or 3600 s at 8 °C. Hydrogen/deuterium exchange was quenched by adding 100 µL of 4 M guanidine HCl, 0.85 M TCEP buffer (final pH was 2.5). The mixture was then subjected to on-column digestion using the protease type XIII/pepsin column. The resultant peptides were trapped and desalted on an ACQUITY UPLC BEH C18 VanGuard pre-column (130 Å, 1.7 µm, 2.1 × 5 mm, 186,003,975, Waters) for 3.5 min at 160 µL/min. Peptides were then eluted from the trap using a 2–30% gradient of acetonitrile (with 0.3% formic acid) over 12.5 min at a flow rate of 150 µL/min and are separated on a 50 × 1 mm C8 column (3 µm, NBA2014015, NovaBioAssays LLC, MA, USA). A UPLC-MS system comprised of a Waters Acquity UPLC coupled to a Q Exactive™ HF Hybrid Quadrupole-Orbitrap Mass Spectrometer (Thermo) was used as described previously^44^. Solvent A was 0.3% formic acid in water. The injection valve and enzyme column and their related connecting tubings were inside a cooling box maintained at 8 °C. The second switching valve, C8 column and their related connecting stainless steel tubings were inside another chilled circulating box maintained at − 6 °C. Peptide identification was done through searching MS/MS data against the CoV-2 RBD sequence using Byonics (Protein Metrics, CA, USA). The mass tolerance for the precursor and product ions were 10 ppm and 0.02 Da, respectively. The mass spectra for deuterated samples were recorded in MS only mode. Raw MS data was processed using HDX WorkBench, software for the analysis of H/D exchange MS data^45^. The deuterium levels were calculated using the average mass difference between the deuterated peptide and its undeuterated form (t_0_).

### Crystallization, data collection, and structure determination

The RBD-7A9 complex at 9.5 mg/mL was treated overnight with EndoH at 4 °C and used in crystallization trials without further purification. Crystals of the space group P3_2_21 were grown at 20 ºC by sitting drop vapor diffusion using a drop ratio of 2:1 protein:reservoir solution. Reservoir solution contained 0.2 M sodium malonate pH 6.0 and 18% PEG 3350. Crystals were cryoprotected in reservoir solution supplemented with 25% glycerol.

X-Ray diffraction data were collected at beamline 17-ID at the Advanced Photon Source. Data were processed using autoPROC^46^ and elliptically truncated using STARANISO^47^. The structure was solved by molecular replacement in Phenix^48^ using the SARS-CoV-2 RBD (PDB 7E7Y; Ref.^49^) and a homology model of 7A9 with the CDRs removed as search models. There was one copy of the complex in the asymmetric unit. The structure was then rebuilt in Coot^50^ and subjected to iterative rounds of refinement and rebuilding using Phenix and Coot. Data processing and refinement statistics are summarized in Supplementary Table 5.

### Analytical ultracentrifugation (AUC)

For AUC experiments, we used the SARS-CoV-2-PreS-Closed Spike protein ectodomain, as described above. This construct contains four stabilizing amino acid substitutions (D614N, A892P, A942P, and V987P), and forms a stable trimer without an artificial trimerization sequence. Samples contained 6 μM 7A9 or 1E4 VHH and/or 1.33 μM CoV-2-PreS-Closed Spike trimer (4 μM monomer) in AUC buffer (50 mM HEPES pH 7.5 + 150 mM NaCl). Samples were mixed and then incubated on a rotating platform at room temperature for 3 h. 0.4 mL of each sample was loaded into the right side and 0.4 mL of AUC buffer was loaded into the left side of an AUC cell containing sapphire windows and a double sector charcoal EPON centerpiece. Balanced cells were loaded into an An50Ti rotor and thermally equilibrated in the chamber of a Beckman Optima AUC under vacuum at 25 °C for 1 h. Samples were centrifuged at 40,000 RPM with a total of 300 scans taken at 40 s intervals using absorption optics set at 280 nm. Values for buffer density, viscosity, and partial specific volumes of the proteins were calculated in Sednterp software^51^. Every 5th scan (60 total) was loaded into the program Sedfit for continuous distribution (c(s)) analysis^52^. Initial fitting of parameters was iteratively performed to minimize r.m.s.d. values. After testing multiple values, the frictional coefficient was held constant at 1.2 for all samples. After optimizing fit parameters using 60 scans, all 300 scans were analyzed and peaks were integrated in Sedfit. All AUC figures were generated using Matplotlib software^53^.

### Size exclusion chromatography/multi-angle light scattering (SEC-MALS)

SEC-MALS experiments were performed on an Agilent 1260 Infinity II HPLC system using a Superose 6 Increase 10/300 GL column (Cytiva) at room temperature. The system was equipped with an Agilent HPLC UV‐detector (280 nm) and Wyatt Technology DAWN Heleos II light‐scattering and T‐rEX refractive index detectors. The mobile phase was composed of PBS buffer supplemented with 0.02% sodium azide and was flowed at a rate of 0.5 mL/min. Protein extinction coefficients were calculated based on sequence using Expasy ProtParam assuming 1-to-1 binding for the Spike-VHH complexes. Refractive index values (dn/dc) were estimated at 0.185 and 0.134 mL/g for protein and glycan moieties, respectively. Samples were mixed and incubated as described in the AUC methods section, and subsequently filtered through a 0.22 μm membrane (Millipore). For each run, 0.1 mL of sample (between 11 and 64 μg total protein) was injected onto the column. Molecular weight values were determined in ASTRA software (Wyatt Technology) using a conjugate analysis. The data was exported, and figures were made using GraphPad Prism software.

### Multimer design

Following domain binning and HDX epitope mapping, the approximate epitope and binding location of many monomeric VHHs against the spike protein were determined. To calculate the length of the desired linker between two VHHs, the distance between the center of the known epitope was measured in both the open and closed state of the spike protein in MOE 2020.09 (Chemical Computing Group). To account for cases where the shortest distance between two sites would result in a clash and to account for the unknown binding orientation of the VHH in relation to the spike protein (where the terminus would be located), an additional buffer distance was added to the initial measurement. Given that an amino acid can cover 3.5–4.0 angstroms in an extended conformation, the measured distance was then converted to a minimum number of amino acids that would be required to bridge such a distance. The resulting number of amino acids was then further converted to the minimum number of linker subunits that could connect the two sites.

### Supplementary Information

Supplementary Figures.

Supplementary Tables.

## Data Availability

Atomic coordinates and structure factors for the reported crystal structure have been deposited with the Protein Data Bank under accession code 8SK5.
