# Supplementary material for: Discovery and multimerization of cross-reactive single-domain antibodies against SARS-like viruses to enhance potency and address emerging SARS-CoV-2 variants
Source: Sci Rep. 2023 Aug 22;13:13668. doi: 10.1038/s41598-023-40919-7 (PMC10444775; doi:10.1038/s41598-023-40919-7)
Supplement: Supplementary file 1 — Supplementary Figures. [file 41598_2023_40919_MOESM1_ESM.pptx]

## Slide 1
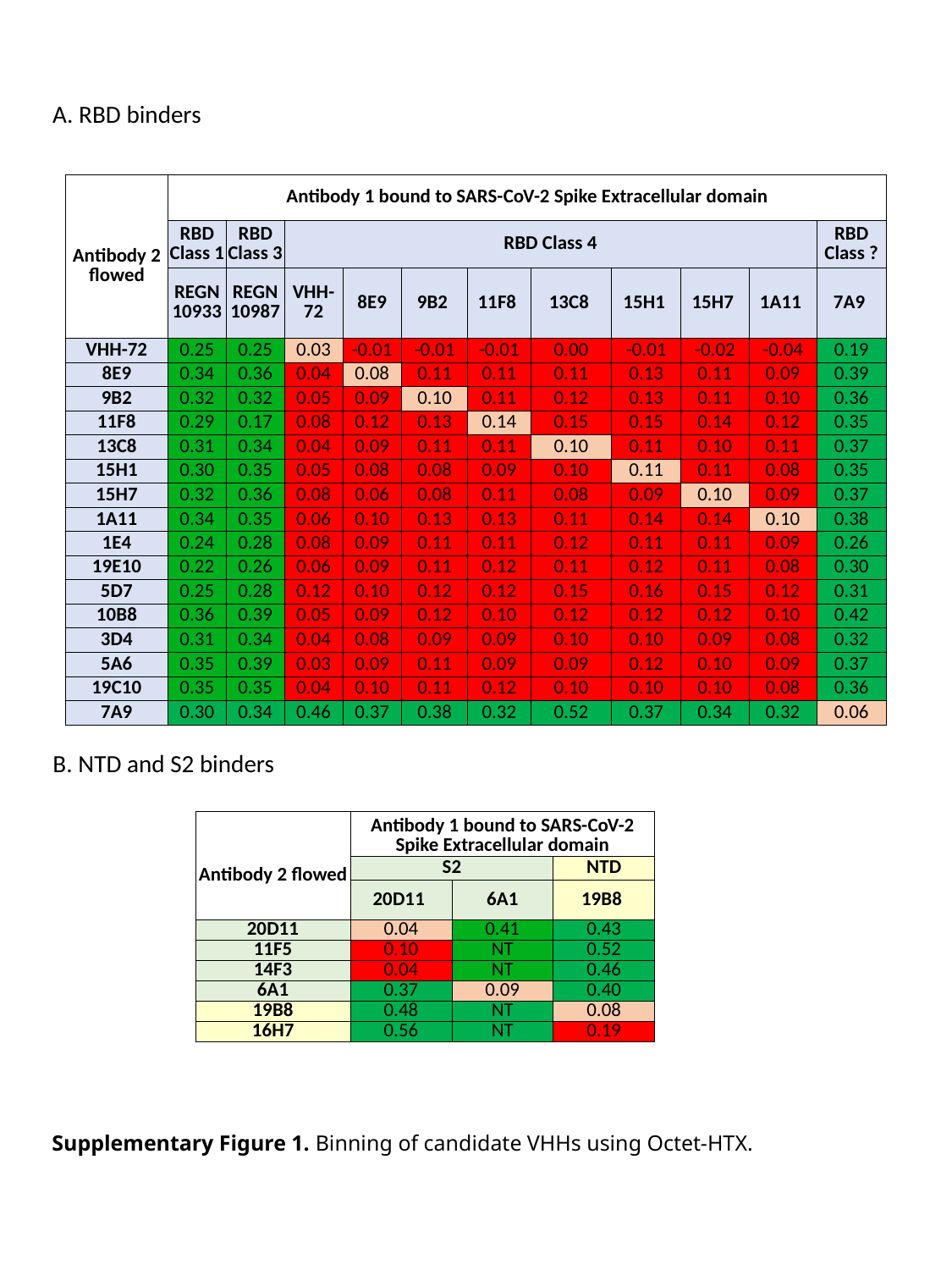

A. RBD binders
| Antibody 2 flowed | Antibody 1 bound to SARS-CoV-2 Spike Extracellular domain | | Antibody 1 bound to biotinylated SARS-CoV-2 Spike ECD | | | | | | | | |
| --- | --- | --- | --- | --- | --- | --- | --- | --- | --- | --- | --- |
| | RBD Class 1 | RBD Class 3 | RBD Class 4 | | | | | | | | RBD Class ? |
| | REGN 10933 | REGN 10987 | VHH-72 | 8E9 | 9B2 | 11F8 | 13C8 | 15H1 | 15H7 | 1A11 | 7A9 |
| VHH-72 | 0.25 | 0.25 | 0.03 | -0.01 | -0.01 | -0.01 | 0.00 | -0.01 | -0.02 | -0.04 | 0.19 |
| 8E9 | 0.34 | 0.36 | 0.04 | 0.08 | 0.11 | 0.11 | 0.11 | 0.13 | 0.11 | 0.09 | 0.39 |
| 9B2 | 0.32 | 0.32 | 0.05 | 0.09 | 0.10 | 0.11 | 0.12 | 0.13 | 0.11 | 0.10 | 0.36 |
| 11F8 | 0.29 | 0.17 | 0.08 | 0.12 | 0.13 | 0.14 | 0.15 | 0.15 | 0.14 | 0.12 | 0.35 |
| 13C8 | 0.31 | 0.34 | 0.04 | 0.09 | 0.11 | 0.11 | 0.10 | 0.11 | 0.10 | 0.11 | 0.37 |
| 15H1 | 0.30 | 0.35 | 0.05 | 0.08 | 0.08 | 0.09 | 0.10 | 0.11 | 0.11 | 0.08 | 0.35 |
| 15H7 | 0.32 | 0.36 | 0.08 | 0.06 | 0.08 | 0.11 | 0.08 | 0.09 | 0.10 | 0.09 | 0.37 |
| 1A11 | 0.34 | 0.35 | 0.06 | 0.10 | 0.13 | 0.13 | 0.11 | 0.14 | 0.14 | 0.10 | 0.38 |
| 1E4 | 0.24 | 0.28 | 0.08 | 0.09 | 0.11 | 0.11 | 0.12 | 0.11 | 0.11 | 0.09 | 0.26 |
| 19E10 | 0.22 | 0.26 | 0.06 | 0.09 | 0.11 | 0.12 | 0.11 | 0.12 | 0.11 | 0.08 | 0.30 |
| 5D7 | 0.25 | 0.28 | 0.12 | 0.10 | 0.12 | 0.12 | 0.15 | 0.16 | 0.15 | 0.12 | 0.31 |
| 10B8 | 0.36 | 0.39 | 0.05 | 0.09 | 0.12 | 0.10 | 0.12 | 0.12 | 0.12 | 0.10 | 0.42 |
| 3D4 | 0.31 | 0.34 | 0.04 | 0.08 | 0.09 | 0.09 | 0.10 | 0.10 | 0.09 | 0.08 | 0.32 |
| 5A6 | 0.35 | 0.39 | 0.03 | 0.09 | 0.11 | 0.09 | 0.09 | 0.12 | 0.10 | 0.09 | 0.37 |
| 19C10 | 0.35 | 0.35 | 0.04 | 0.10 | 0.11 | 0.12 | 0.10 | 0.10 | 0.10 | 0.08 | 0.36 |
| 7A9 | 0.30 | 0.34 | 0.46 | 0.37 | 0.38 | 0.32 | 0.52 | 0.37 | 0.34 | 0.32 | 0.06 |
B. NTD and S2 binders
| Antibody 2 flowed | Antibody 1 bound to SARS-CoV-2 Spike Extracellular domain | | |
| --- | --- | --- | --- |
| | S2 | | NTD |
| | 20D11 | 6A1 | 19B8 |
| 20D11 | 0.04 | 0.41 | 0.43 |
| 11F5 | 0.10 | NT | 0.52 |
| 14F3 | 0.04 | NT | 0.46 |
| 6A1 | 0.37 | 0.09 | 0.40 |
| 19B8 | 0.48 | NT | 0.08 |
| 16H7 | 0.56 | NT | 0.19 |
Supplementary Figure 1. Binning of candidate VHHs using Octet-HTX.

## Slide 2
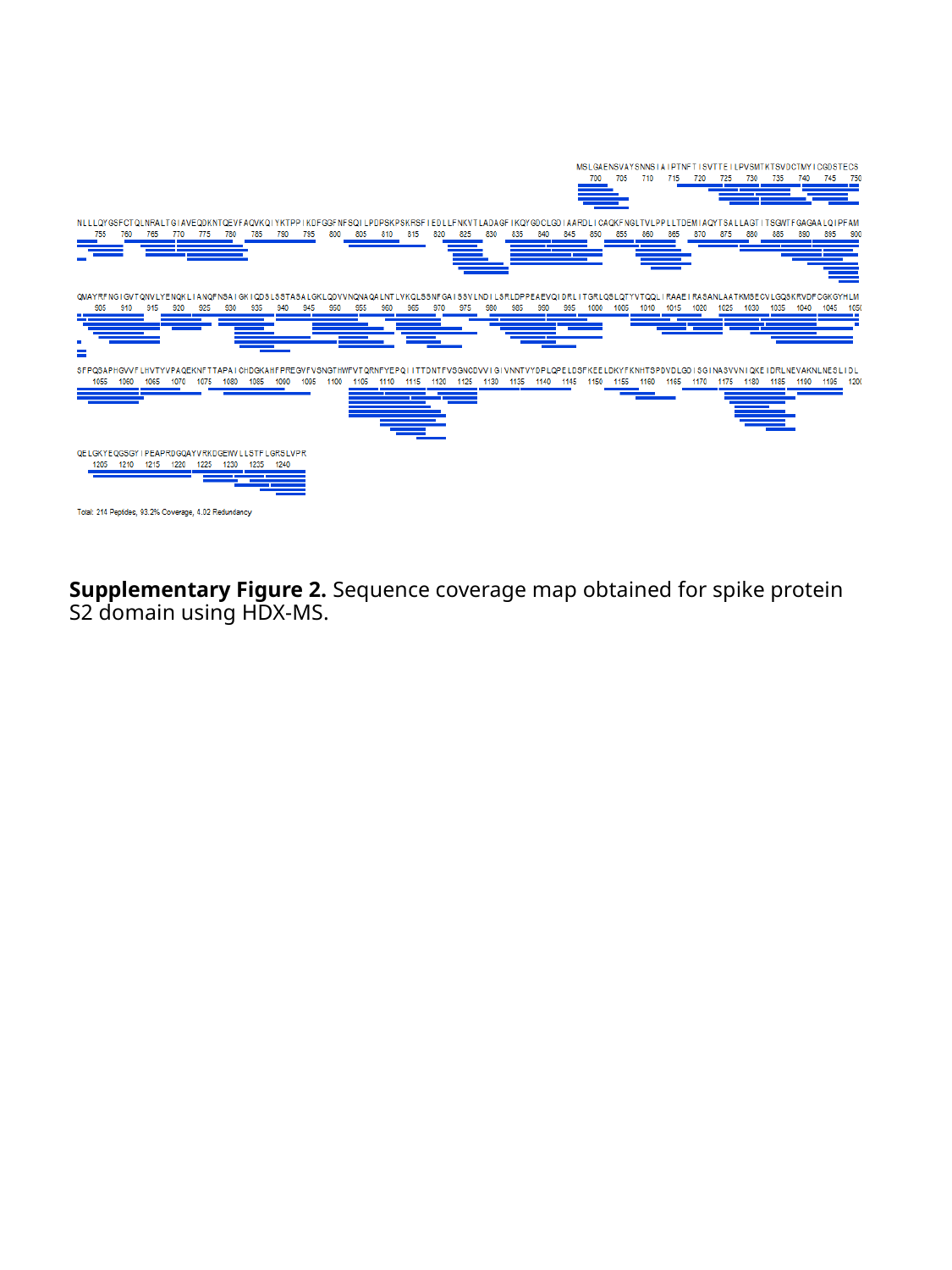

Supplementary Figure 2. Sequence coverage map obtained for spike protein S2 domain using HDX-MS.

## Slide 3
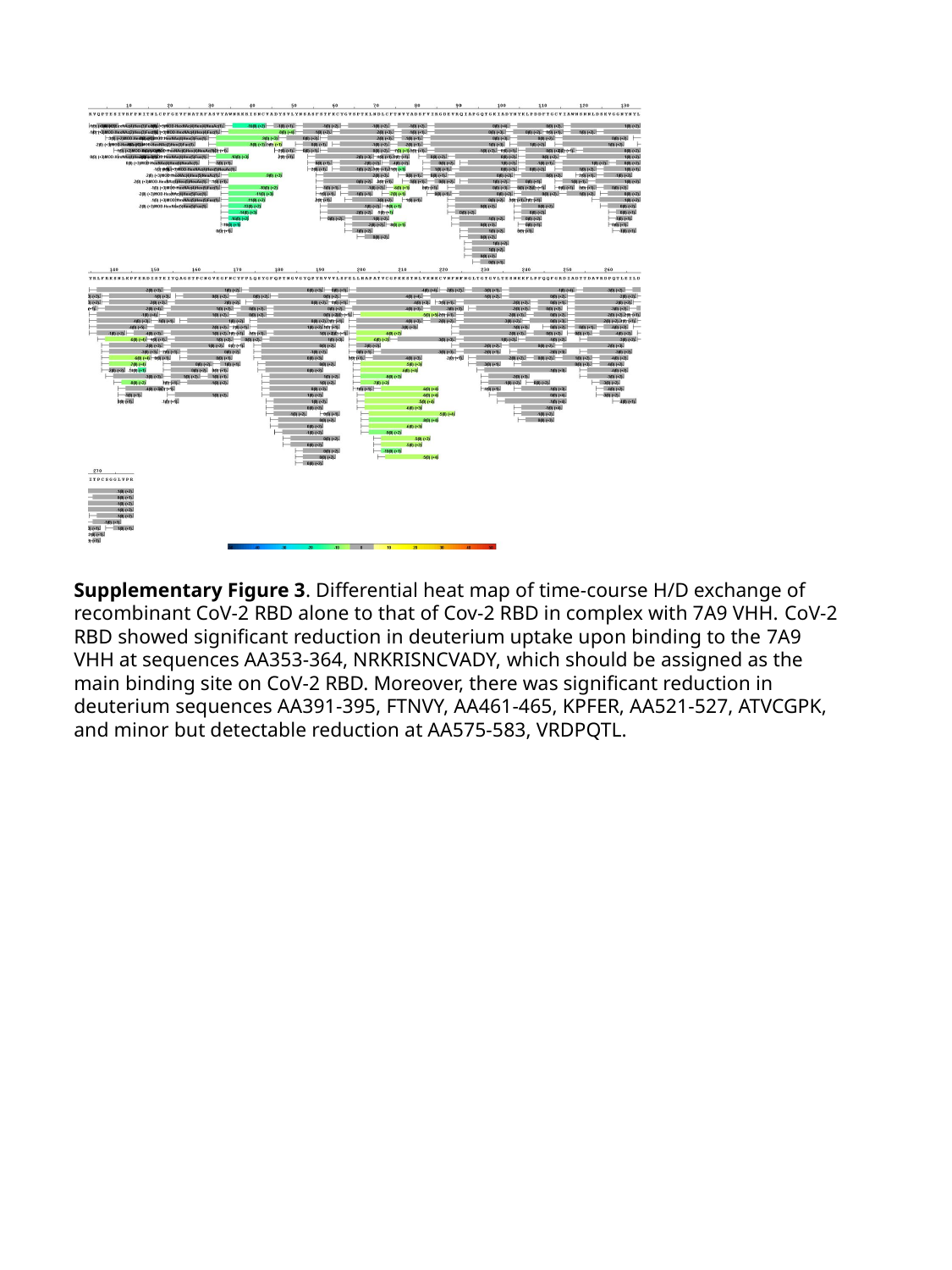

Supplementary Figure 3. Differential heat map of time-course H/D exchange of recombinant CoV-2 RBD alone to that of Cov-2 RBD in complex with 7A9 VHH. CoV-2 RBD showed significant reduction in deuterium uptake upon binding to the 7A9 VHH at sequences AA353-364, NRKRISNCVADY, which should be assigned as the main binding site on CoV-2 RBD. Moreover, there was significant reduction in deuterium sequences AA391-395, FTNVY, AA461-465, KPFER, AA521-527, ATVCGPK, and minor but detectable reduction at AA575-583, VRDPQTL.

## Slide 4
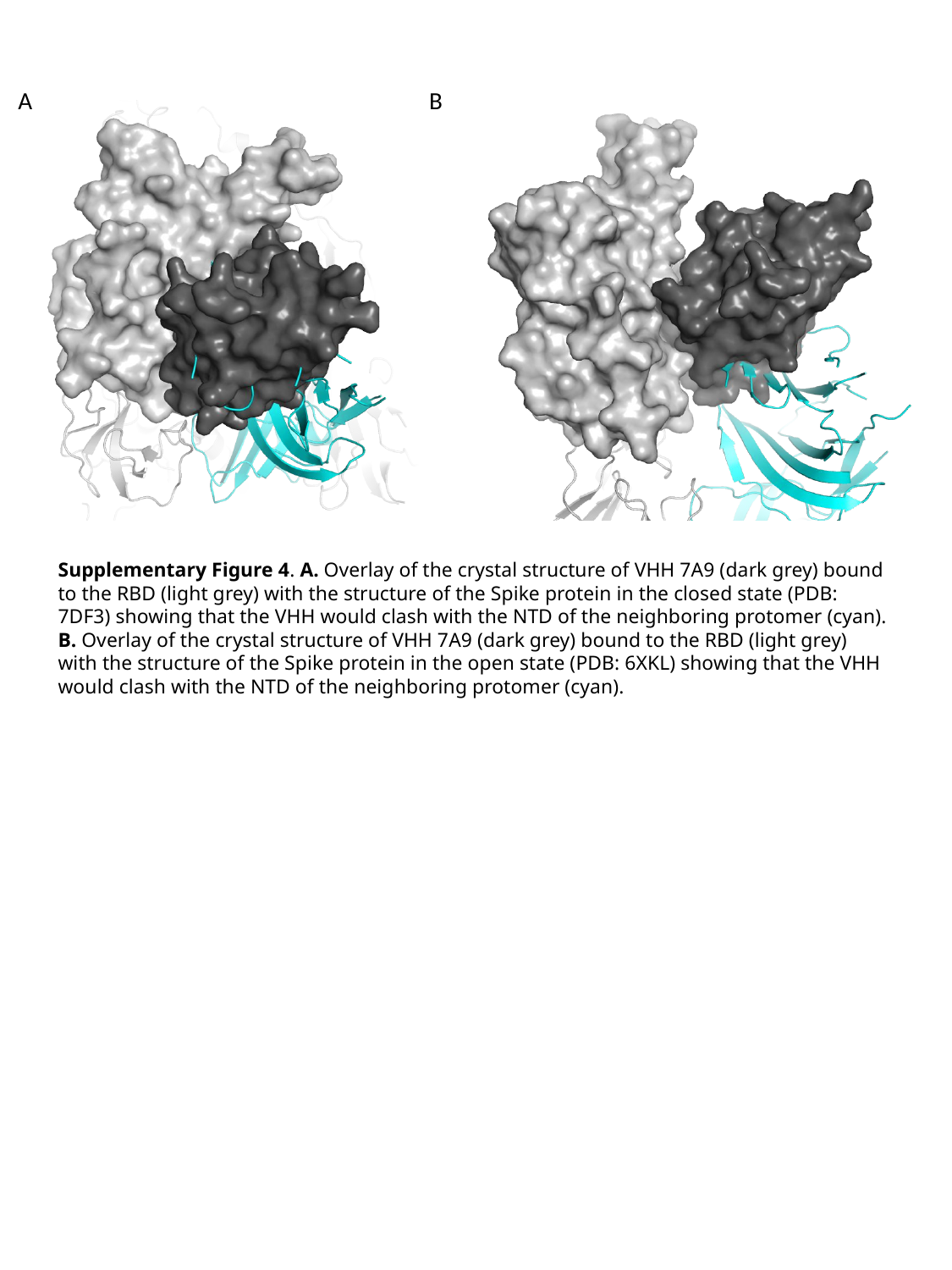

A
B
Supplementary Figure 4. A. Overlay of the crystal structure of VHH 7A9 (dark grey) bound to the RBD (light grey) with the structure of the Spike protein in the closed state (PDB: 7DF3) showing that the VHH would clash with the NTD of the neighboring protomer (cyan). B. Overlay of the crystal structure of VHH 7A9 (dark grey) bound to the RBD (light grey) with the structure of the Spike protein in the open state (PDB: 6XKL) showing that the VHH would clash with the NTD of the neighboring protomer (cyan).

## Slide 5
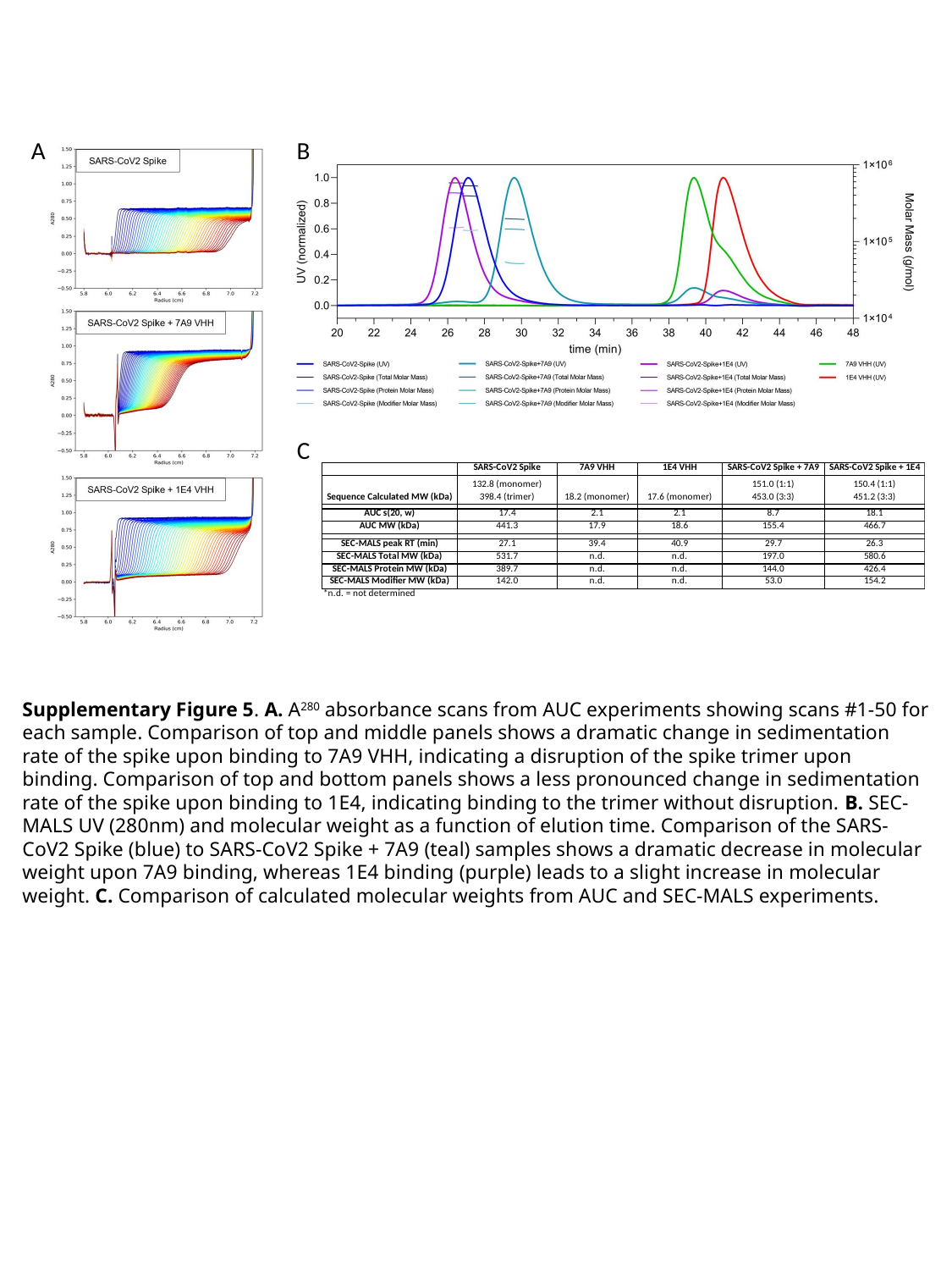

A
B
C
Supplementary Figure 5. A. A280 absorbance scans from AUC experiments showing scans #1-50 for each sample. Comparison of top and middle panels shows a dramatic change in sedimentation rate of the spike upon binding to 7A9 VHH, indicating a disruption of the spike trimer upon binding. Comparison of top and bottom panels shows a less pronounced change in sedimentation rate of the spike upon binding to 1E4, indicating binding to the trimer without disruption. B. SEC-MALS UV (280nm) and molecular weight as a function of elution time. Comparison of the SARS-CoV2 Spike (blue) to SARS-CoV2 Spike + 7A9 (teal) samples shows a dramatic decrease in molecular weight upon 7A9 binding, whereas 1E4 binding (purple) leads to a slight increase in molecular weight. C. Comparison of calculated molecular weights from AUC and SEC-MALS experiments.

## Slide 6
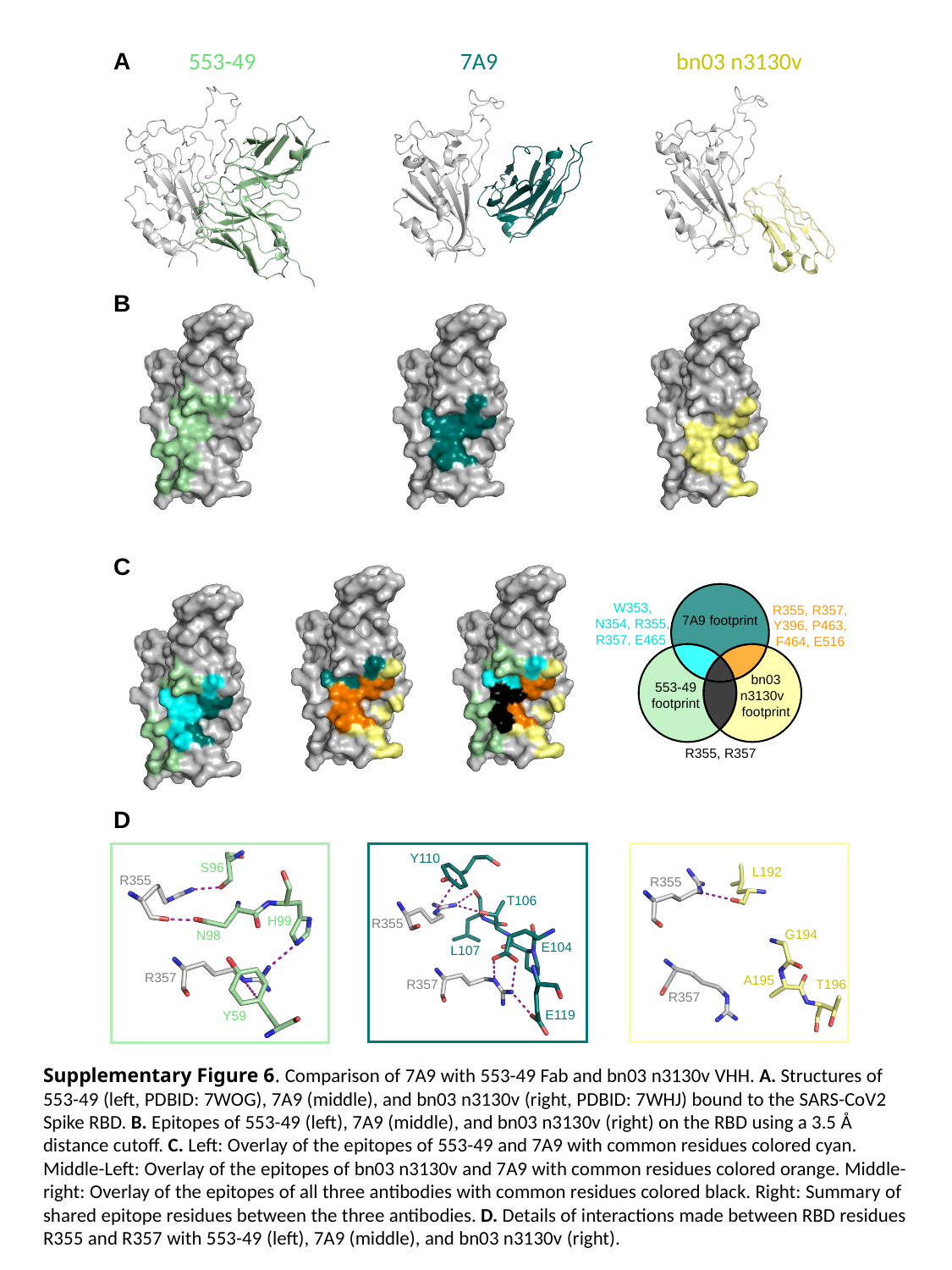

A
7A9
553-49
bn03 n3130v
B
C
7A9 footprint
W353, N354, R355, R357, E465
R355, R357, Y396, P463, F464, E516
R355, R357
bn03 n3130v footprint
553-49 footprint
D
S96
R355
H99
N98
R357
Y59
Y110
T106
R355
E104
L107
R357
E119
L192
R355
G194
A195
T196
R357
Supplementary Figure 6. Comparison of 7A9 with 553-49 Fab and bn03 n3130v VHH. A. Structures of 553-49 (left, PDBID: 7WOG), 7A9 (middle), and bn03 n3130v (right, PDBID: 7WHJ) bound to the SARS-CoV2 Spike RBD. B. Epitopes of 553-49 (left), 7A9 (middle), and bn03 n3130v (right) on the RBD using a 3.5 Å distance cutoff. C. Left: Overlay of the epitopes of 553-49 and 7A9 with common residues colored cyan. Middle-Left: Overlay of the epitopes of bn03 n3130v and 7A9 with common residues colored orange. Middle-right: Overlay of the epitopes of all three antibodies with common residues colored black. Right: Summary of shared epitope residues between the three antibodies. D. Details of interactions made between RBD residues R355 and R357 with 553-49 (left), 7A9 (middle), and bn03 n3130v (right).
