# Supplementary material for: Discovery and multimerization of cross-reactive single-domain antibodies against SARS-like viruses to enhance potency and address emerging SARS-CoV-2 variants
Source: Sci Rep. 2023 Aug 22;13:13668. doi: 10.1038/s41598-023-40919-7 (PMC10444775; doi:10.1038/s41598-023-40919-7)
Supplement: Supplementary file 2 — Supplementary Tables. [file 41598_2023_40919_MOESM2_ESM.docx]

Supplementary Table 1: ELISA binding of VHH-Fcs (EC50 values)

| **Clone Name** | **Binding EC50 (nM)** | | | | | | | | | | |
| --- | --- | --- | --- | --- | --- | --- | --- | --- | --- | --- | --- |
|  | **WT Cov2** | **Alpha \| B.1.1.7** | **Beta \| B.1.351** | **Gamma \| P.1** | **Delta \| B.1.617.2** | **Omicron \|BA1.1** | **Omicron \| BA.2** | **WT Cov1** | **MERS** | **OC43** | **HKU1** |
| 8E9 | 0.08 | 0.06 | 0.10 | 0.15 | 0.06 | 0.60 | 0.60 | 0.08 | NB |  |  |
| 9B2 | 0.10 | 0.06 | 0.07 | 0.05 | 0.06 | 0.18 | NB | 0.12 | NB |  |  |
| 11F8 | 0.19 | 0.08 | 0.21 | 0.14 | 0.11 | NB | NB | 0.18 | NB |  |  |
| 13C8 | 0.09 | 0.11 | 0.24 | 0.23 | 0.18 | NB | NB | 0.10 | NB |  |  |
| 15H1 | 0.14 | 0.05 | 0.10 | 0.09 | 0.10 | NB | NB | 0.06 | NB |  |  |
| 15H7 | 0.05 | 0.05 | 0.07 | 0.06 | 0.06 | NB | NB | 0.08 | NB |  |  |
| 1A11 | 0.18 | 0.04 | 0.62 | 0.24 | 0.20 | 0.64 | 0.64 | 0.05 | NB |  |  |
| 1E4 | 0.04 | 0.62 | 0.03 | 0.03 | 0.06 | NB | NB | 0.04 | NB |  |  |
| 19E10 | 0.03 | 0.06 | 0.05 | 0.03 | 0.04 | 0.09 | 0.09 | 0.03 | NB |  |  |
| 5D7 | 0.11 | 0.74 | 0.22 | 0.09 | 0.06 | 0.22 | 0.22 | 0.04 | NB |  |  |
| 10B8 | 1.35 | 0.05 | 0.03 | 1.11 | 0.85 | 2.81 | 2.81 | 0.13 | NB |  |  |
| 3D4 | 0.13 | 0.11 | 0.09 | 0.09 | 0.08 | NB | NB | 0.12 | NB |  |  |
| 5A6 | 0.14 | 0.11 | 0.15 | 0.10 | 0.08 | NB | NB | 0.09 | NB |  |  |
| 19C10 | 0.06 | 0.18 | 0.04 | 0.14 | 0.10 | NB | NB | 0.08 | NB |  |  |
| 7A9 | 0.16 | 0.13 | 0.13 | 0.12 | 0.17 | 0.19 | 0.19 | 0.06 | NB |  |  |
| 19B8 | 0.06 | 0.63 | 0.26 | 0.17 | 0.08 | 0.04 | 0.04 | 0.04 | NB |  |  |
| 16H7 | 0.08 | 0.06 | 0.18 | 0.04 | 0.62 | 0.03 | 0.51 | 0.18 | NB |  |  |
| 20D11 | 0.62 | 0.03 | 0.06 | 0.56 | 0.69 | 0.53 | 0.53 | 0.04 | NB |  |  |
| 11F5 | 0.12 | 0.05 | 0.06 | 0.05 | 0.05 | 0.04 | 0.04 | 0.09 | NB |  |  |
| 14F3 | 0.05 | 0.03 | 0.08 | 0.03 | 0.03 | 0.03 | 0.03 | 0.05 | NB |  |  |
| 6A1 | 0.44 | 0.20 | 0.20 | 0.18 | 0.27 | 0.20 | 0.20 | 0.10 | 0.08 | 0.08 | 1.60 |
| S3_44 | WB | WB | WB | WB | WB | WB | WB | WB | NB | WB |  |
| S3_29 | WB | WB | WB | WB | WB | WB | WB | WB | NB | WB |  |

NB No binding

WB Weakly binding, EC50 not calculated

Supplementary Table 2: FACS binding of VHH-Fc constructs (EC50 values)

| **Clone Name** | **Binding EC50 (nM)** | | |
| --- | --- | --- | --- |
|  | **WT Cov2** | **WT Cov1** | **MERS** |
| 8E9 | 0.31 | 0.17 | NB |
| 9B2 | 0.21 | 0.13 | NB |
| 11F8 | 0.08 | 0.09 | NB |
| 13C8 | 0.22 | 0.11 | NB |
| 15H1 | 0.25 | 0.14 | NB |
| 15H7 | 0.28 | 0.14 | NB |
| 1A11 | 0.13 | 0.06 | NB |
| 1E4 | 0.06 | 0.06 | NB |
| 19E10 | 0.14 | 0.08 | NB |
| 5D7 | 0.12 | 0.09 | NB |
| 10B8 | 0.19 | 0.14 | NB |
| 3D4 | 0.15 | 0.12 | NB |
| 5A6 | 0.43 | 0.21 | NB |
| 19C10 | 0.17 | 0.13 | NB |
| 7A9 | 0.08 | 0.05 | NB |
| 19B8 | 0.04 | 0.06 | NB |
| 16H7 | 0.31 | 0.10 | NB |
| 20D11 | 0.08 | 0.10 | NB |
| 11F5 | 0.04 | 0.05 | NB |
| 14F3 | 0.06 | 0.09 | NB |
| 6A1 | 0.14 | 0.19 | 0.13 |
| S3_44 | 0.08 | 0.09 | 0.11 |
| S3_29 | 0.03 | 0.04 | 0.05 |

NB No binding

WB Weakly binding, EC50 not calculated

Supplementary Table 3: Apparent affinities of VHH and VHH-Fc constructs to SARS-CoV2 spike protein. Since VHH-Fc molecules are able to bind bivalently, values listed are affected by avidity and are not monovalent affinity values.

| **Clone Name** | **Affinity KD (M)** | |
| --- | --- | --- |
|  | **VHH** | **VHH-Fc** |
| 8E9 | 5.7E-09 | <2.0E-10 |
| 9B2 | 6.1E-10 | <2.0E-10 |
| 11F8 | 9.5E-10 | <2.0E-10 |
| 13C8 | 1.2E-08 | <2.0E-10 |
| 15H1 | 6.0E-10 | <2.0E-10 |
| 15H7 | 3.3E-10 | <2.0E-10 |
| 1A11 | <2.0E-10 | <2.0E-10 |
| 1E4 | 2.5E-10 | <2.0E-10 |
| 19E10 | 7.3E-10 | <2.0E-10 |
| 5D7 | 1.5E-09 | <2.0E-10 |
| 10B8 | <2.0E-10 | <2.0E-10 |
| 3D4 | <2.0E-10 | <2.0E-10 |
| 5A6 | 4.6E-10 | <2.0E-10 |
| 19C10 | <5.0E-08 | <2.0E-10 |
| 7A9 | 2.2E-10 | <2.0E-10 |
| 19B8 | 8.7E-08 | 1.1E-09 |
| 16H7 | 2.3E-07 | 3.0E-09 |
| 20D11 | 2.2E-09 | <2.0E-10 |
| 11F5 | 8.0E-09 | <2.0E-10 |
| 14F3 | 3.0E-09 | <2.0E-10 |
| 6A1 | >5.0E-07 | 1.6E-09 |
| S3_44 | NB | NB |
| S3_29 | NB | NB |

NB No binding

Supplementary Table 4: Pseudovirus neutralization potency of VHH-Fc constructs

| **Clone Name** | **Domain** | **Neutralization potency IC50 (nM)** | | | | | | |
| --- | --- | --- | --- | --- | --- | --- | --- | --- |
|  |  | **WT Cov2** | **WT Cov1** | **MERS** | **Alpha \| B.1.1.7** | **Beta \| B.1.351** | **Gamma \| P.1** | **Omicron \|BA1.1** |
| 8E9 | RBD | 12.4 | 2.94 | NN |  |  |  | 429 |
| 9B2 | RBD | 1.93 | 0.19 | NN | 22 | 4.2 | 21 | 52 |
| 11F8 | RBD | 1.07 | 206.8 | NN |  |  |  | NN |
| 13C8 | RBD | 6.69 | 4.98 | NN |  |  |  |  |
| 15H1 | RBD | 5.04 | 12.31 | NN |  |  |  |  |
| 15H7 | RBD | 1.33 | 1.71 | NN |  |  |  |  |
| 1A11 | RBD | 0.57 | 0.18 | NN | 6.6 | 4.1 | 6.4 | 42 |
| 1E4 | RBD | 0.31 | 0.06 | NN | 2.2 | 0.58 | 2.6 |  |
| 19E10 | RBD | 1.53 | 0.12 | NN | 7 | 3.1 | 5.9 | 309 |
| 5D7 | RBD | 6.52 | 220.3 | NN |  |  |  | 221 |
| 10B8 | RBD | 0.68 | 0.09 | NN | 0.38 | 0.23 | 0.21 | 4.1 |
| 3D4 | RBD | 1.22 | 0.40 | NN | 1.5 | 1.1 | 0.59 |  |
| 5A6 | RBD | 8.57 | 6.30 | NN | 26 | 54 | 3.1 |  |
| 19C10 | RBD | 1.49 | 0.45 | NN | 0.85 | 0.66 | 0.8 |  |
| 7A9 | RBD | 110 | WN | NN |  |  |  | 44 |
| 19B8 | NTD | 53.45 | 42.03 | NN |  |  |  | 47 |
| 16H7 | NTD | NN | NN | NN |  |  |  |  |
| 20D11 | S2 | NN | NN | NN |  |  |  |  |
| 11F5 | S2 | NN | NN | NN |  |  |  |  |
| 14F3 | S2 | NN | NN | NN |  |  |  |  |
| 6A1 | S2 | NN | NN | NN |  |  |  |  |
| S3_44 | S2 | NN | NN | NN |  |  |  |  |
| S3_29 | S2 | NN | NN | NN |  |  |  |  |

NN Non-neutralizing

WN Weakly-neutralizing, IC50 not calculated

Supplementary Table 5: Crystallographic data collection and refinement statistics

|  | RBD-7A9  (PDB 8SK5) |
| --- | --- |
| **Data collection** |  |
| Space group | P 3_2_ 2 1 |
| Cell dimensions |  |
| *a*, *b*, *c* (Å) | 93.90, 93.91, 121.39 |
| α, β, γ (°) | 90, 90, 120 |
| Resolution (Å) | 81.32 - 2.35 (isotropic)  81.32 - 2.01 (ellipsoidal) (2.22 - 2.01)* |
| *R*_sym_ or *R*_merge_ | 0.069 (0.959) |
| *I* / σ*I* | 18.3 (1.7) |
| Completeness (%) | 95.4 (74.8) |
| Redundancy | 9.9 (6.9) |
|  |  |
| **Refinement** |  |
| Resolution (Å) | 36.23 - 2.01 (anisotropic) |
| No. reflections | 29572 (1476) |
| *R*_work_ / *R*_free_ | 0.1832 / 0.2109 |
| No. atoms |  |
| Protein | 2537 |
| Ligand/ion | 38 |
| Water | 157 |
| *B*-factors |  |
| Protein | 47.39 |
| Ligand/ion | 87.07 |
| Water | 47.4 |
| R.m.s. deviations |  |
| Bond lengths (Å) | 0.003 |
| Bond angles (°) | 0.583 |

*Values in parentheses are for highest-resolution shell.

[AU: Equations defining various *R*-values are standard and hence are no longer defined in the footnotes.]

[AU: Ramachandran statistics should be in Methods section at the end of Refinement subsection.]

[AU: Wavelength of data collection, temperature and beamline should all be in Methods section.]

Supplementary Table 6: Pseudovirus neutralization potency of multimer vs cocktail

| **Multimer Clone Name** | **WT CoV2** | | | **WT CoV1** | | |
| --- | --- | --- | --- | --- | --- | --- |
|  | **Multimer IC50 (nM)** | **Cocktail IC50 (nM)** | **Fold improvement** | **Multimer IC50 (nM)** | **Cocktail IC50 (nM)** | **Fold improvement** |
| **7A9**-20aa(GS)-**10B8**-20aa(GS)-**19B8** | 2.79 | 0.75 | 0.3 |  |  |  |
| **10B8**-20aa(GS)-**19B8**-50aa(GAS)-**20D11** | 1.04 | 2.08 | 2 |  |  |  |
| **7A9**-20aa(GS)-**1E4**-50aa(GAS)-**6A1** | 1.99 | 3.72 | 1.9 |  |  |  |
| **1E4**-20aa(GS)-**7A9**-50aa(GAS)-**6A1** | 2.88 | 3.72 | 1.3 |  |  |  |
| **10B8**-20aa(GS)-**7A9**-50aa(GAS)-**S3-29** | 0.99 | 5.9 | 6 |  |  |  |
| **10B8**-20aa(GS)-**19B8**-50aa(GAS)-**S3-29** | 0.31 | 1.97 | 6.4 |  |  |  |
| **10B8**-20aa(GS)-**7A9**-20aa(GS)-**19B8** | 1.7 | 2.6 | 1.5 |  |  |  |
| **10B8**-20aa(GS)-**19B8**-50aa(GAS)-**6A1** | 1.58 | 2.75 | 1.7 |  |  |  |
| **7A9**-20aa(GS)-**10B8**-50aa(GAS)-**S3-29** | 2.5 | 5.9 | 2.4 | 0.52 | 0.19 | 0.37 |
| **7A9**-20aa(GS)-**19B8**-50aa(GAS)-**S3-29** | 5.7 | 246 | 43 | 1.3 | 344 | 265 |
| **7A9**-20aa(GS)-**19B8**-90aa(PAS)-**20D11** | 29 | 136 | 4.7 | 11 | 947 | 86 |
| **7A9**-20aa(GS)-**19B8**-90aa(PAS)-**6A1** | 69 | 233 | 3.4 | 6.81 | 1205 | 177 |
| **19B8**-50aa(GAS)-**S3-29**-50aa(GAS)-**7A9** | 1.14 | 87 | 76 | 6.09 | 431 | 71 |
| **19B8**-50aa(GAS)-**S3-29**-50aa(GAS)-**S3_29** | 15 | 436 | 29 | 95 | 1156 | 12 |
| **S3_29**-50aa(GAS)-**S3-29**-50aa(GAS)-**7A9** | 53 | 162 | 3 | 104 | 1092 | 10 |
| **S3_29**-50aa(GAS)-**19B8**-90aa(PAS)-**6A1** | 20 | 763 | 39 | 16 | 1197 | 73 |
| **19E10**-50aa(GAS)-**S3-29**-50aa(GAS)-**7A9** | <1 | 31 | - | 1.77 | 335 | 189 |
| **19E10**-50aa(GAS)-**19B8**-90aa(PAS)-**6A1** | 5.9 | 27 | 4.6 | 0.65 | 298 | 456 |
| **19E10**-90aa(PAS)-**11F5**-90aa(PAS)-**6A1** | 3.2 | 37 | 12 | 259 | 272 | 1.05 |
| **19E10**-50aa(GAS)-**S3-29**-50aa(GAS)-**19E10** | <1 | 36 | - | 0.38 | 202 | 531 |

| **Trimer Clone Name** | **WT CoV2** | | | **WT CoV1** | | |
| --- | --- | --- | --- | --- | --- | --- |
|  | **Multimer IC50 (nM)** | **Cocktail IC50 (nM)** | **Fold improvement** | **Multimer IC50 (nM)** | **Cocktail IC50 (nM)** | **Fold improvement** |
| **1E4**-20aa(GS)-**1E4**-20aa(GS)-**1E4** | 0.46 | 12 | 26 | 0.044 | 42 | 955 |
| **7A9**-20aa(GS)-**7A9**-20aa(GS)-**7A9** | 671 | 788 | 1.2 | 830 | N/A | - |
| **10B8**-20aa(GS)-**10B8**-20aa(GS)-**10B8** | 1.18 | 1.18 | 1 | 0.15 | 0.19 | 1.2 |

| < 2 |
| --- |
| 2-5 |
| > 5 |

RBD-binding VHHs

NTD-binding VHHs

S2-binding VHHs
